# PANoptosis-related prognostic signature predicts overall survival of cutaneous melanoma and provides insights into immune infiltration landscape

**DOI:** 10.1038/s41598-023-35462-4

**Published:** 2023-05-25

**Authors:** Wei Wang, Qingde Zhou, Lan Lan, Xinchang Xu

**Affiliations:** 1 Department of Pharmacy, Hangzhou Third People’s Hospital, Affiliated Hangzhou Dermatology Hospital, Zhejiang University School of Medicine, West Lake Road 38, Hangzhou, 310009 People’s Republic of China; 2 Department of Dermatology, Affiliated Hangzhou Dermatology Hospital, Zhejiang University School of Medicine, West Lake Road 38, Hangzhou, 310009 People’s Republic of China

**Keywords:** Cancer, Computational biology and bioinformatics, Biomarkers, Oncology

## Abstract

Cutaneous melanoma (CM) is a highly malignant tumor originating from melanocytes, and its metastasis and recurrence are the major causes of death in CM patients. PANoptosis is a newly defined inflammatory programmed cell death that crosstalk pyroptosis, apoptosis, and necroptosis. PANoptosis participates in the regulation of tumor progression, especially the expression of PANoptosis related genes (PARGs). Although pyroptosis, apoptosis, and necroptosis have received attention in CM, respectively, the link between them remains elusive. Therefore, this study aimed to investigate the potential regulatory role of PANoptosis and PARGs in CM and the relationship among PANoptosis, PARGs and tumor immunity. We identified 3 PARGs associated with prognosis in CM patients by The Cancer Genome Atlas. Risk model and nomogram were established. Enrichment analysis of differentially expressed genes indicated that CM was immune-related. Subsequent analyses indicated that prognosis-related PARGs were associated with immune scores and infiltration of immune cells in CM patients. In addition, immunotherapy and drug sensitivity results indicated an association between prognosis-related PARGs and drug resistance in CM patients. In conclusion, PARGs play a key role in the progression of tumors in CM patients. PARGs can be used not only for risk assessment and OS prediction in CM patients, but also reflect the immune landscape of CM patients, which can provide a novel reference for individualized tumor treatment.

## Introduction

Among all skin tumors, cutaneous melanoma (CM) is the skin tumor with the highest mortality rate^1^. According to the GLOBOCAN 2020 cancer report prepared by the International Agency for Research on Cancer, there will be nearly more than 300,000 new cancer cases and 60,000 new cancer deaths in 2020 alone^2^. Notably, the mortality rate of CM is four times higher than that of non-melanoma skin tumors^2^. The 5-year survival rate of CM patients remains unsatisfactory due to the fact that CM is a highly metastatic malignancy^3^. Therefore, early detection and diagnosis of CM can be of great help in the treatment of CM^4^. Nowadays, research on the diagnosis and treatment of CM still worthwhile, especially the discovery of some reliable prognostic biomarkers will be crucial^5^.

The imbalance between apoptosis and proliferation was the direct cause of tumorigenesis^6^. Notably, alterations in the apoptotic program can also affect tumor response to drugs^7,8^. Therefore, understanding the new programmed death pathways is essential for tumor prevention, diagnosis, and treatment. PANoptosis is a recently newly defined inflammatory programmed cell death that is based on an inflammatory vesicle complex (PANoptosome)^9^. It has been shown that PANoptosis, which contains molecules which associated with pyroptosis, apoptosis, and necroptosis, can crosstalk them and influence their relationship with each other^10–14^.

The key role of PANoptosis in many tumors has been reported, such as colorectal cancer^15^, gastric cancer^16^, and adrenocortical cancer^17^. It has also been shown that pyroptosis, apoptosis, and necroptosis are all closely related to CM^18,19^. However, current studies of PANoptosis in CM tend to focus on only one or two of them, and the joint roles of all three of them have not been reported. Therefore, the discovery of relevant biomarkers of PANoptosis in CM is crucial. In conclusion, the better understanding of the role of PANoptosis could provide new insights into the diagnosis and targeted therapy of CM.

In this study, PANoptosis-related genes (PARGs) associated with OS in CM patients were identified by analyzing datasets downloaded from the Cancer Genome Atlas database (TCGA). The risk model was constructed based on prognosis-related PARGs and the predictive power of the model was evaluated. Enrichment analysis of differentially expressed genes (DEGs) was used to further understand the differences in biological function between CM patients with different risks. The immune landscape of CM patients was assessed using a combination of immune algorithms. In addition, we analyzed the differences in immunotherapy response and drug sensitivity between patients with different risks. Overall, this study may provide new insights into the diagnosis and targeted therapy of CM.

## Materials and methods

### Data collection and preliminary processing

The transcriptome matrix and relevant clinical information of CM patients involved in this study were downloaded from the Cancer Genome Atlas database (TCGA) (https://portal.gdc.cancer.gov/). Transcriptomes without survival time information will not be considered and a total of 454 CM samples were finally available for subsequent analysis (Table 1 and Supplementary Table 1). The transcriptome of each CM sample was merged for annotation by perl script^20^. Age, sex, grade, stage, and TMN stage were obtained from the relevant clinical information downloaded from TCGA. The data involved in this study were downloaded from public databases and did not require ethics committee approval or written informed consent from patients.

**Table 1:** Clinical information on the samples involved in this study.

Clinical	Group	Cohorts	Training cohorts	Test cohorts
Age	≤ 65	296	206	90
	> 65	158	112	46
Gender	Female	172	122	50
	Male	282	196	86
Fustat	Alive	236	157	79
	Dead	218	161	57
Stage	0	6	4	2
	1	76	51	25
	2	136	99	37
	3	169	118	51
	4	22	15	7
	Unknown	45	31	14
T	0	23	15	8
	1	41	24	17
	2	76	52	24
	3	89	68	21
	4	147	107	40
	Unknown	78	52	26
M	0	405	281	124
	1	23	16	7
	Unknown	26	21	5
N	0	225	159	66
	1	73	56	17
	2	49	32	17
	3	54	33	21
	Unknown	53	38	15

### Identification of prognosis-associated PARGs and construction of risk models

A total of 14 PARGs were extracted based on previous studies, and these genes are mainly components of the PANoptosome^9^. Univariate Cox regression analysis was used to assess the association of PARGs with CM. The least absolute shrinkage and selection operator (LASSO) algorithm was used to further screen PARGs associated with OS of CM. PARGs with independent prediction of CM prognosis were identified by multivariate Cox regression analysis, and risk models were constructed based on prognosis-related PARGs with the formula: Risk score = ∑_i_ = Coef_i_ ∗ (expression of PANRG_i_). The CM sample was divided into low-risk and high-risk groups according to the median risk score. Kaplan–Meier survival curves were used to assess the differences between OS of CM patients with different risks.

### Internal validation of the risk model

To validate the predictive ability of the risk model, 454 CM samples were divided into a training cohort and a test cohort according to a 7:3 ratio and risk scores were calculated. There were 318 samples in the training cohort and 136 samples in the test cohort. Afterwards, the samples in each cohort were divided into low risk and high risk groups based on the median risk scores of each cohort. In each cohort, Kaplan–Meier survival curves were used to analyze the difference in OS between patients in the low- and high-risk groups.

### Independent prognostic analysis of risk models and clinical characteristics

Prognostic independence of risk model and clinical characteristics was assessed by univariate and multivariate Cox regression analysis (performed by the R package “survival”). Kaplan–Meier survival curves were used to demonstrate differences in OS of CM patients with different clinical characteristics. The nomogram based on risk models and clinical characteristics were constructed by the R package "rms", and predictive accuracy was assessed by calibration plots and decision curves. The predicted survival probabilities of all variables were analyzed and visualized by the R package “pROC” and the R package “ggplot2”.

### Screening and biological function enrichment for DEGs

The “limma” R package was used to evaluate and screen DEGs between different risk patient groups with a |Fold Change|≥ 2 and a *p* value < 0.05. Kyoto Encyclopedia of Genes and Genomes (KEGG) analysis and Gene Ontology (GO) analysis were performed with the “clusterProfiler” R package to assess the enrichment of pathways and biological functions involved in DEGs^21^. Visualization of KEGG enrichment results by the “ggplot2” R package.

### Immune landscape and drug sensitivity analysis

Multiple immune algorithms were used to assess the immune landscape of the samples in our study. The ESTIMATE algorithm can estimate stromal and immune cells and provide an ESTIMATE score. The CIBERSORT algorithm can assess the proportion of 22 immune cells in each sample. The single sample gene set enrichment analysis (ssGSEA) allows the assessment of 23 immune cells as well as immune function^22^. Tumor immune dysfunction and rejection (TIDE) scores for CM patients were calculated through the TIDE database (http://tide.dfci.harvard.edu/login/). Anti-tumor drug response prediction was performed in CM patients based on the Genomics of Drug Sensitivity in Cancer (GDSC) database, as reflected by half maximal inhibitory concentration (IC50)^23^. Differences in IC50 between different groups of CM patients were analyzed and visualized by the “ggplot2” R package.

### Consensus clustering analysis

To further clarify the relationship between PARGs and prognosis, CM patients were distinguished into several subgroups based on prognosis-related PARGs. And a multifaceted comparative analysis of different subgroups will be performed, including Kaplan–Meier survival analysis, immune infiltration landscape, and immunotherapy response.

### Statistical analysis

Statistical analyses involved in this study were performed using R software (version 4.1.2), and Wilcoxon rank sum test was used to analyze the differences between the two groups, with *p value < 0.05 as the threshold value to determine whether there was a statistical difference.

## Results

### Construction of a risk model for CM based on PARGs prognostic signature

**Figure 1:**
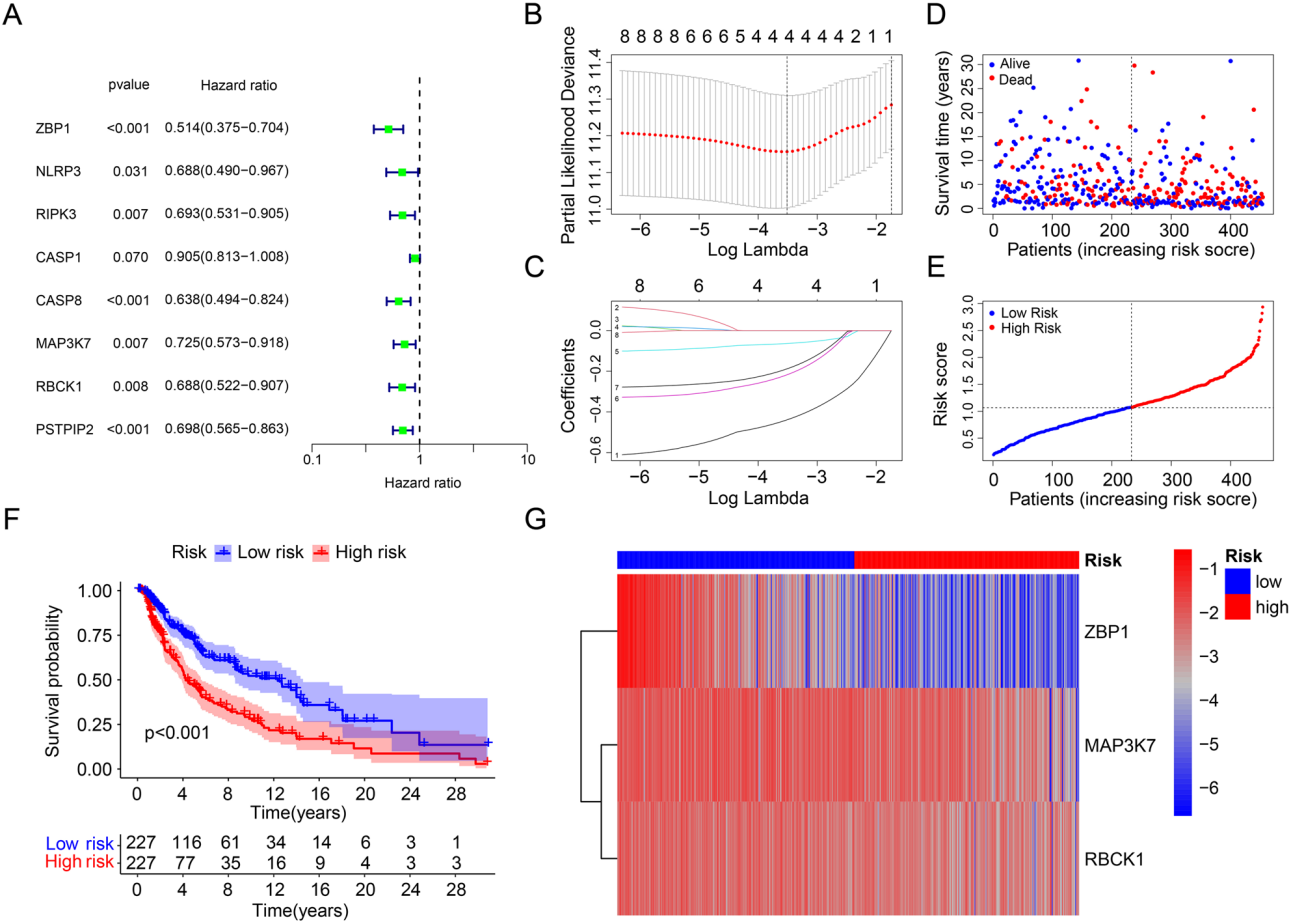
Construction of risk model based on prognosis-related PARGs and analysis of OS in CM patients. (**A**) Results of univariate cox regression analysis of PARGs. HR = 1: No impact; HR < 1: reduced risk, positively correlated with survival time; HR > 1: Increased risk, negatively correlated with survival time. Minimum λ (**B**) and optimal coefficients (**C**) of prognostic PARGs based on Lasso regression analysis. (**D**) Scatter plot of risk scores for CM patients. (**E**) Ranking of risk scores of CM patients. (**F**) Results of Kaplan–Meier survival curve analysis based on risk model. (**G**) Heat map of the expression of prognosis-related PARGs in CM patients. *p < 0.05; **p < 0.01; ***p < 0.001.

To illustrate the relationship between PARGs and CM, a new risk model based on PARGs was constructed to predict the prognosis of CM. As shown in Fig. 1A, 8 PARGs were determined to be associated with OS of CM by univariate Cox regression analysis. Further analysis by the LASSO showed that 4 PARGs were able to predict the OS of CM (Fig. 1B,C). By multivariate Cox regression analysis, 3 PARGs with independent predictive power were finally identified for modeling. Patients with CM were ranked according to median risk score and divided into low- and high-risk groups. The scatter plot results showed that patients in the low-risk group tended to have higher OS (Fig. 1D,E). Kaplan–Meier survival curve analysis showed that patients in different risk groups had different OS (Fig. 1F). Notably, patients in the low-risk group had a higher OS than those in the high-risk group. The expression heat map of the 3 prognosis-related PARGs in the low-risk and high-risk group is shown in Fig. 1G. The results indicated that these 3 prognosis-related PARGs (ZBP1, MAP3K7, and RBCK1) were highly expressed in the low-risk group and less expressed in the high-risk group.

### Validation of PARGs prognostic signature in training and test cohorts

**Figure 2:**
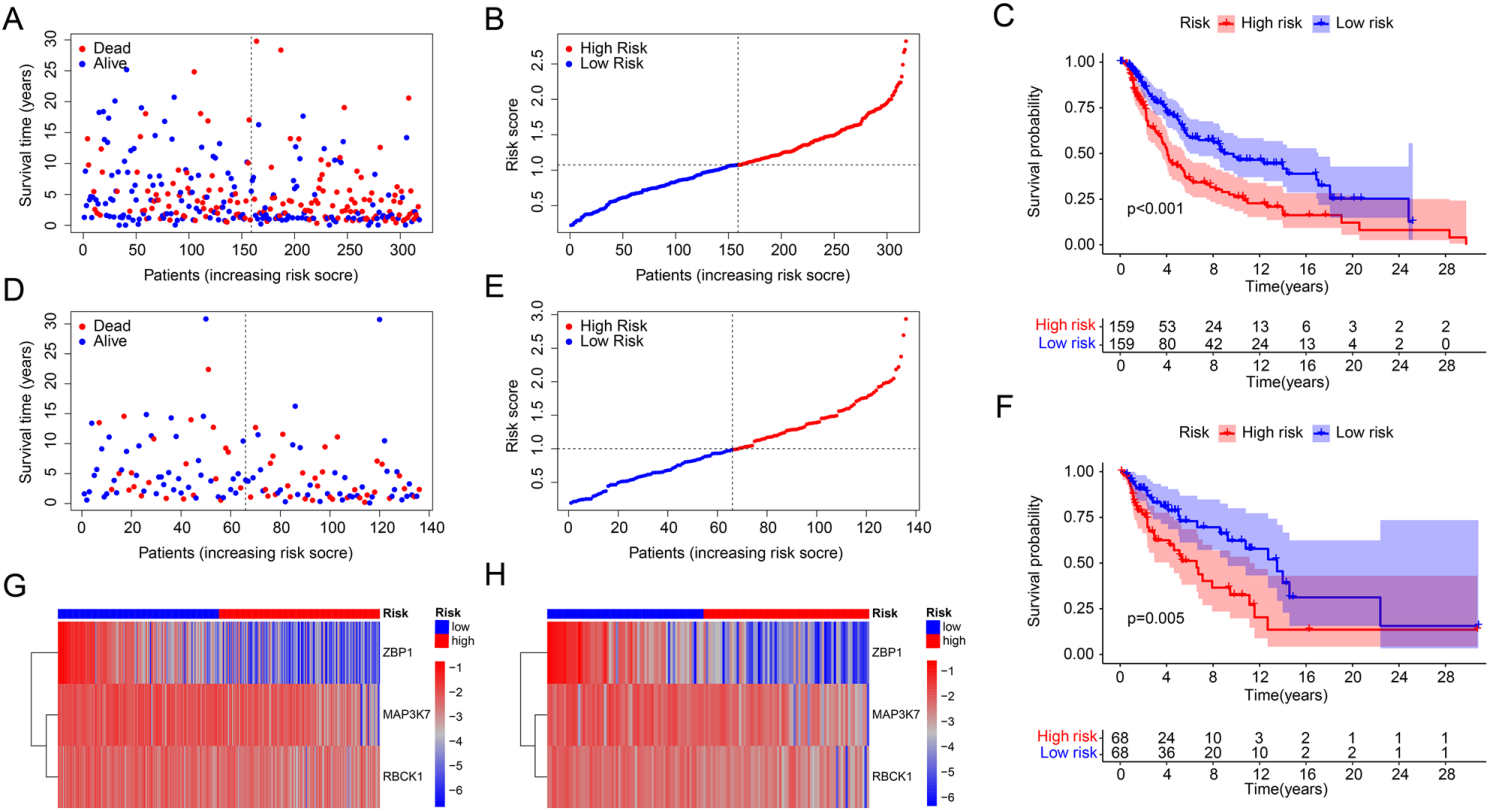
Validation results of PARG prognostic signature in the training and test cohorts. (**A**) Scatter plot of risk scores for CM patients in training cohorts. (**B**) Ranking of risk scores of CM patients in training cohorts. (**C**) Results of Kaplan–Meier survival curve analysis based on risk model in training cohorts. (**D**) Scatter plot of risk scores for CM patients in test cohorts. (**E**) Ranking of risk scores of CM patients in test cohorts. (**F**) Results of Kaplan–Meier survival curve analysis based on risk model in test cohorts. Heat map of the expression of prognosis-related PARGs in CM patients in training (**G**) and test cohorts (**H**). *p < 0.05; **p < 0.01; ***p < 0.001.

Internal validation was performed to assess the accuracy and independence of the PARGs prognostic signature in predicting the prognosis of CM patients.CM patients were randomly divided into a training cohort and a test cohort in a ratio of 7:3. Same as before, patients were divided into low- and high-risk groups according to the PARGs prognostic signature in both cohorts. As shown in Fig. 2A,B,D,E, scatter plots indicate that risk score was negatively correlated with survival time for patients in both the training and test cohorts. The Kaplan–Meier survival curve results indicate that in both cohorts, patients with low risk score had higher OS than patients with high risk score (Fig. 2C,F). The heat map showed that the expression of ZBP1, MAP3K7 and RBCK1 was significantly higher in the low-risk group (Fig. 2G,H). These results suggest that risk model construction based on the prognostic characteristics of PARGs can accurately assess the prognosis of CM patients.

### Risk model based on the PARGs prognostic signature was an independent prognosis indicator

**Figure 3:**
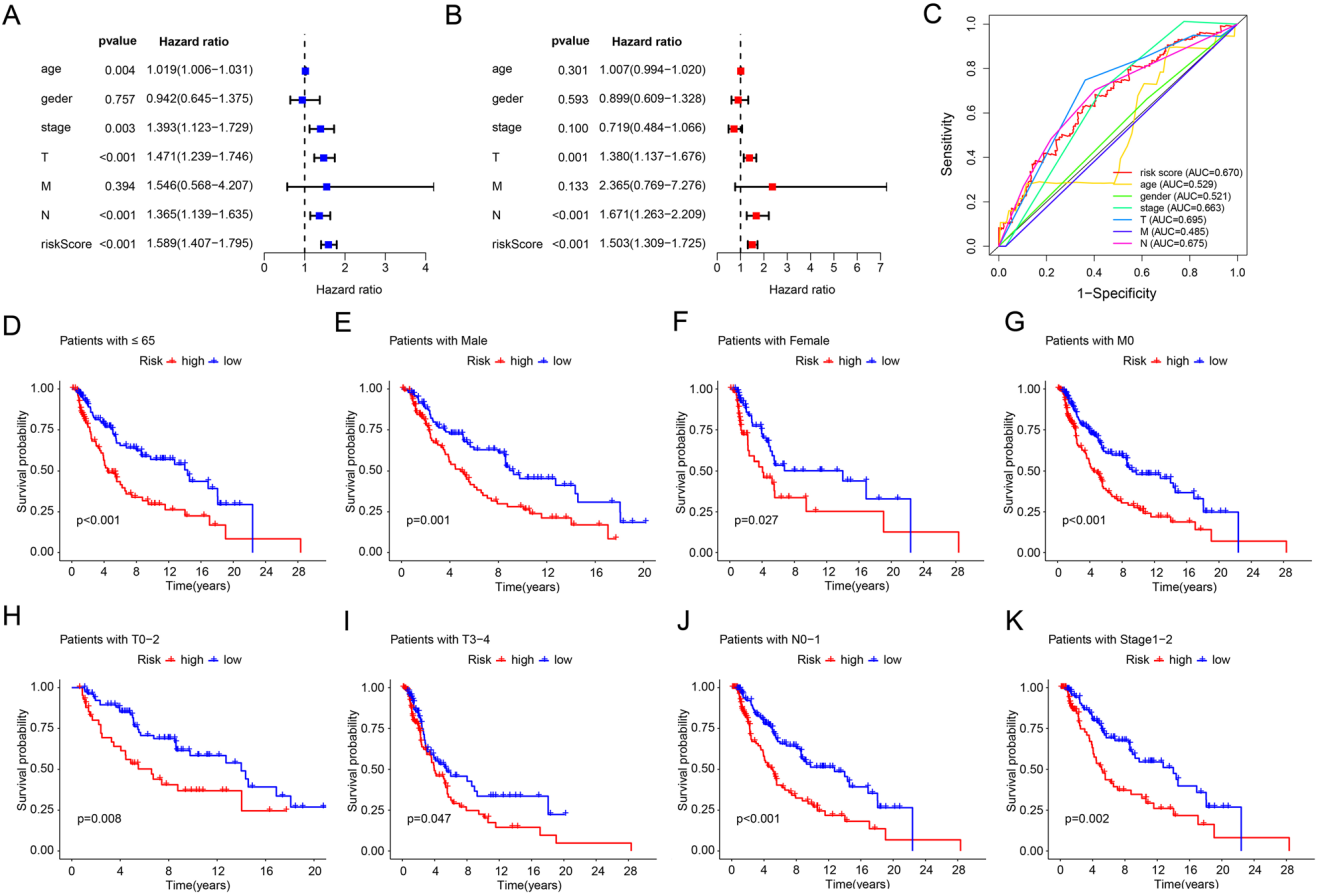
Results of independent prognostic analysis and clinical correlation analysis of risk models based on prognostic characteristics of PARGs. Results of (**A**) univariate Cox regression analysis and (**B**) multivariate Cox regression analysis based on risk score, clinical characteristics with OS. HR = 1: No impact; HR < 1: reduced risk, positively correlated with survival time; HR > 1: Increased risk, negatively correlated with survival time. (**C**) ROC results for predictive accuracy of risk score and clinical characteristics. Correlation analysis based on PARGs prognostic characteristics with different clinical characteristics: (**D**) Age ≤ 65; (**E**) Male; (**F**) Female; (**G**) M 0; (**H**) T I-II; (**I**) T III-IV; (**J**) N 0–1; (**K**) Stage I-II. *p < 0.05; **p < 0.01; ***p < 0.001.

Univariate and multivariate Cox regression analyses were used to assess the independence of risk scores predicting prognosis based on the PARGs prognostic signature. Univariate Cox regression analysis showed that age (hazard ratio (HR) = 1.019, *p* = 0.004), stage (HR = 1.393, *p* = 0.003), T (HR = 1. 471, *p* < 0.001), N (HR = 1.365, *p* < 0.001) and risk score (HR = 1.589, *p* < 0.001) were associated with the OS rate of CM were strongly correlated (Fig. 3A). The results of multivariate Cox regression analysis showed that T (HR = 1.380, *p* = 0.001), N (HR = 1.671, *p* < 0.001) and risk score (HR = 1.503, *p* < 0.001) were independent prognostic indicators of CM (Fig. 3B). The ROC curve showed that the AUC of risk score was 0.670, indicating that the PARGs prognostic signature were satisfactory stability (Fig. 3C).

To further assess the prognostic value of the PARGs prognostic signature under different clinical conditions, we grouped CM patients according to their clinical characteristics. Based on the PARGs prognostic signature, CM patients in the same clinical conditions were divided into low-risk and high-risk groups before Kaplan–Meier survival analysis. As shown in Fig. 3D–K, in some clinical conditions such as female, male, age ≤ 65 years, M0, T0-2, T3-4, N0-1 and Stage1-2 in the low-risk score of patients had significantly higher OS rates than those in the high-risk group. In contrast, OS rates were similar in some CM patients with clinical conditions of age > 65 years, M1, N2-3, and Stage3-4 (data not shown).

**Figure 4:**
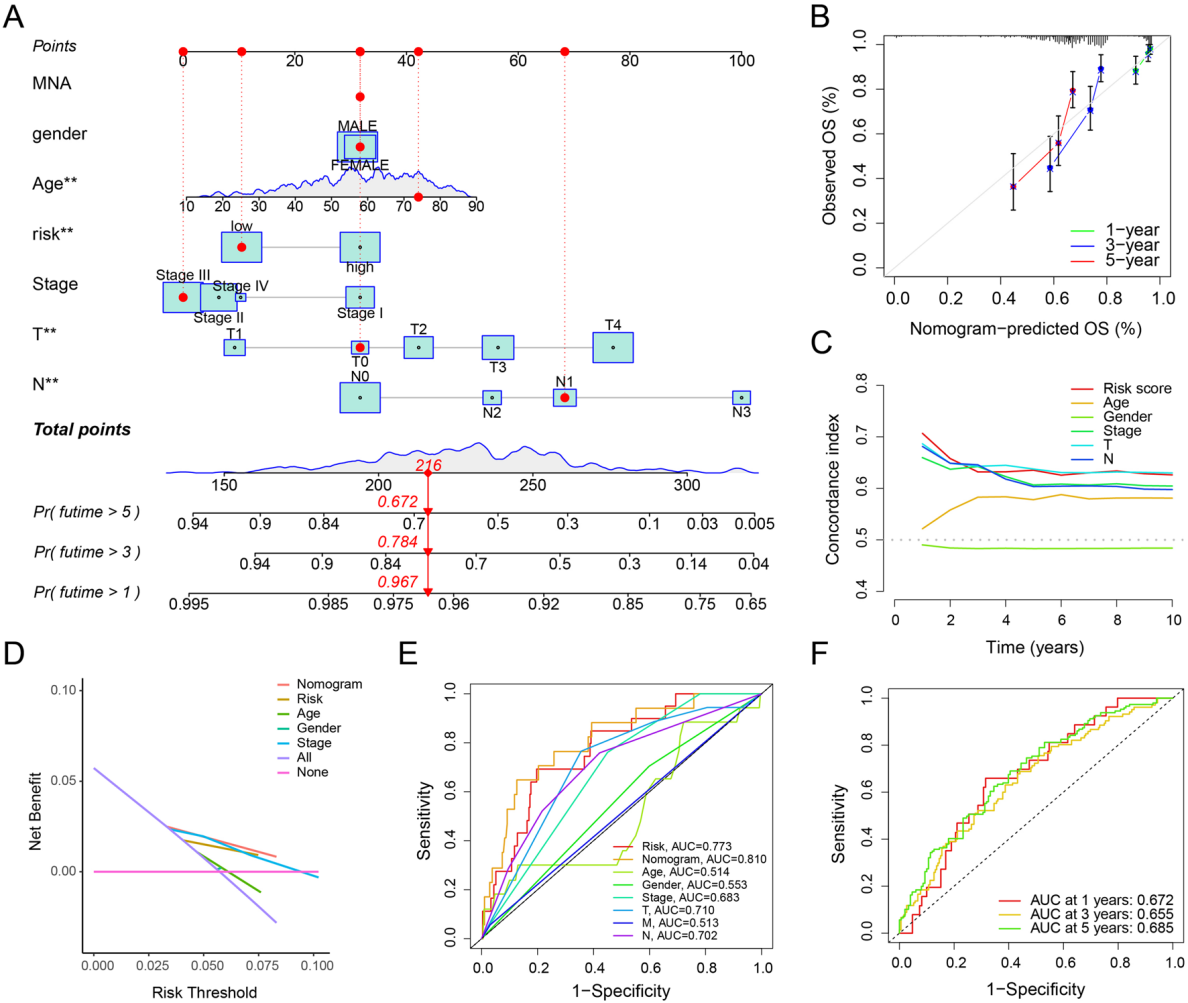
Nomogram construction and prediction accuracy assessment. (**A**) Nomogram construction based on the PARGs prognostic signature and clinical characteristics. (**B**) Calibration curves comparing the accuracy of nomogram predicted OS with the actual OS. Concordance index of the PARGs prognostic signature and clinical characteristics (**C**) and decision curve plots (**D**). (**E**) ROC results for predictive accuracy of nomogram, risk score and clinical characteristics. (**F**) ROC curves based on nomogram predicted at 1, 3 and 5 years. *p < 0.05; **p < 0.01; ***p < 0.001.

A novel nomogram model was subsequently developed to accurately predict the 1-, 3-, and 5-year OS rates of CM patients based on the PARGs prognostic signature and clinicopathological features. As shown in Fig. 4A, there are corresponding scoring criteria for each feature, by scoring each feature, the total score of the sample can be obtained, and the OS can be obtained according to the total score. The calibration curves showed that the 1-, 3-, and 5-year survival times predicted by nomogram showed satisfactory agreement with the actual OS rates of CM patients (Fig. 4B). In addition, the nomogram had a higher concordance index (C-index) and decision curve (Fig. 4C–E) compared to other clinical factors. The time-dependent ROC showed AUCs of 0.672, 0.655, and 0.685 for 1, 3, and 5 years, respectively (Fig. 4F). In conclusion, these results suggest that risk scores based on PARGs can accurately assess the prognosis of CM patients relative to clinicopathological characteristics.

### Enrichment analysis of DEGs

**Figure 5:**
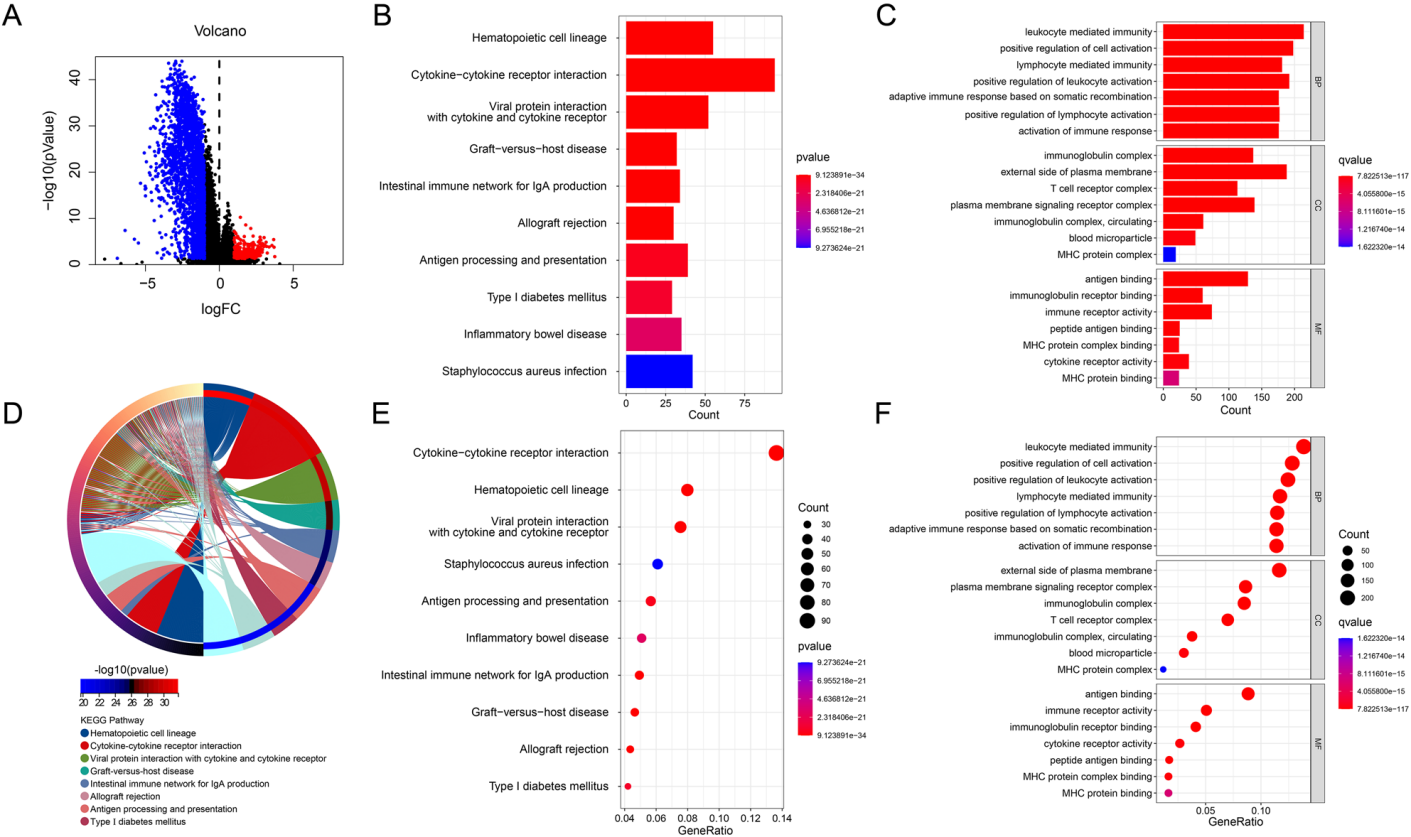
Results of the enrichment analysis of DEGs. (**A**) Volcano plot of DEGs. Bar-plot of the results of KEGG enrichment analysis (**B**) and GO enrichment analysis (**C**). (**D**) Circle-plot of the results of KEGG enrichment analysis. Bubble-plot of the results of KEGG enrichment analysis (**E**) and GO enrichment analysis (**F**). *p < 0.05; **p < 0.01; ***p < 0.001.

KEGG enrichment and GO enrichment were used to investigate the potential molecular mechanisms of DEGs in low and high risk groups. Volcano plot results of DEGs showed that most DEGs were downregulated in the high risk group (Fig. 5A). KEGG analysis as shown in Fig. 5B,D,E and the result showed that Cytokine-cytokine receptor interaction, Antigen processing and presentation, etc. were significantly enriched in DEGs (Fig. 5D). GO enrichment analysis demonstrated a high correlation between DEGs and immunity (Fig. 5C,F). Lymphocyte mediated immunity, positive regulation of leukocyte activation, positive regulation of lymphocyte activation, activation of immune response were enriched in the BP fraction. Immunoglobulin complex, T cell receptor complex, circulating immunoglobulin complex, and immunoglobulin receptor complex were enriched in the CC fraction. Immunoglobulin complex, circulating, immunoglobulin receptor binding, immune receptor activity, cytokine receptor activity were enriched in the MF fraction. These findings suggest that immune-related signaling pathways may mediate the role of PARGs in CM tumorigenesis.

### Assessment and correlation analysis of the immune landscape based on PARGs prognostic signature

**Figure 6:**
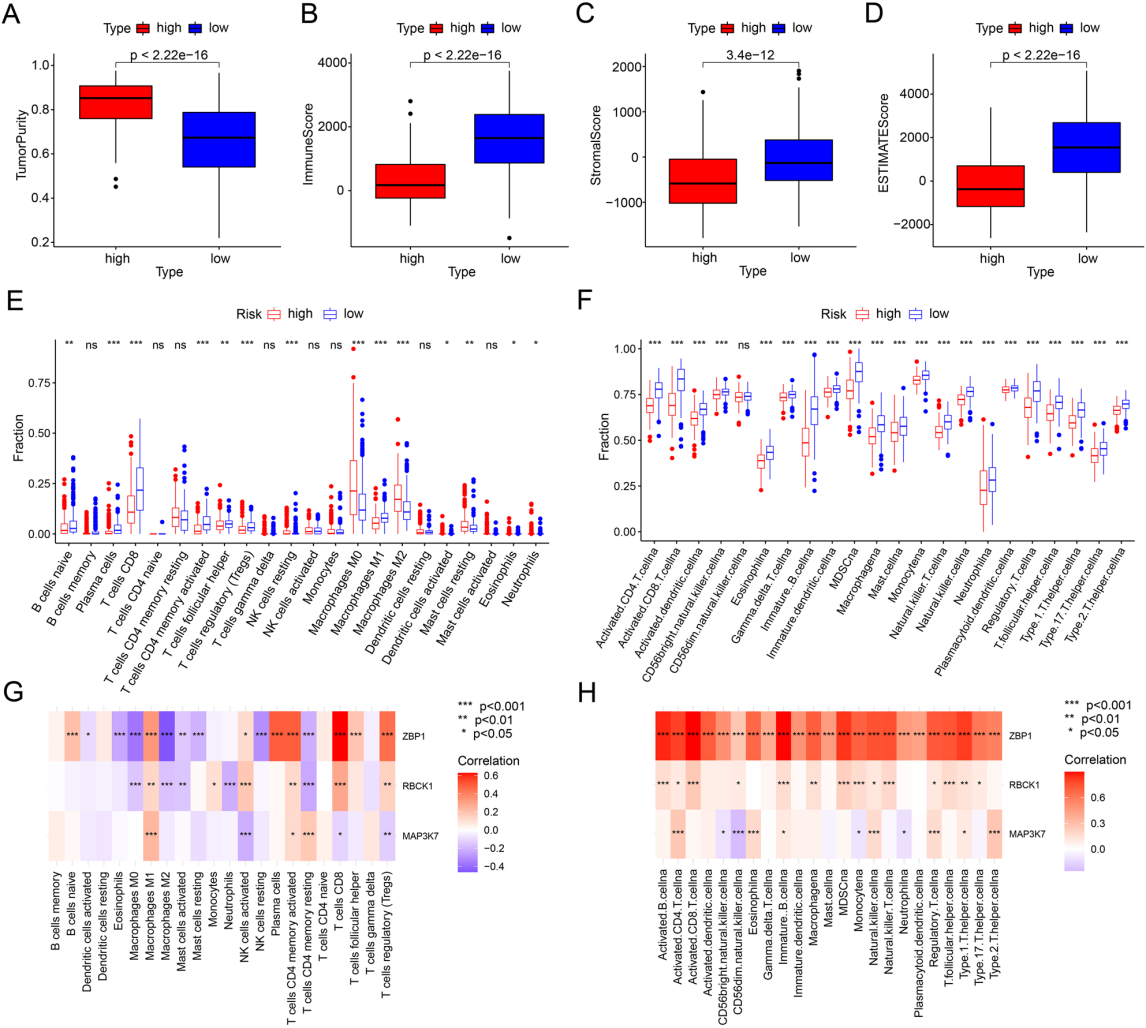
The assessment and correlation analysis result of the immune landscape based on PARGs prognostic signature. (**A**–**D**) Tumor purity results as well as immune, stromal and ESTIMATE scores by ESTIMATE algorithm. (**E**) Proportion of immune cells of type 22 in CM patients of different risk groups by CIBERSORT. (**F**) Proportion of immune cells of type 23 in CM patients of different risk groups by ssGSEA. (**G**, **H**) Correlation results of prognosis-related PARGs with immune cells. *p < 0.05; **p < 0.01; ***p < 0.001.

**Figure 7:**
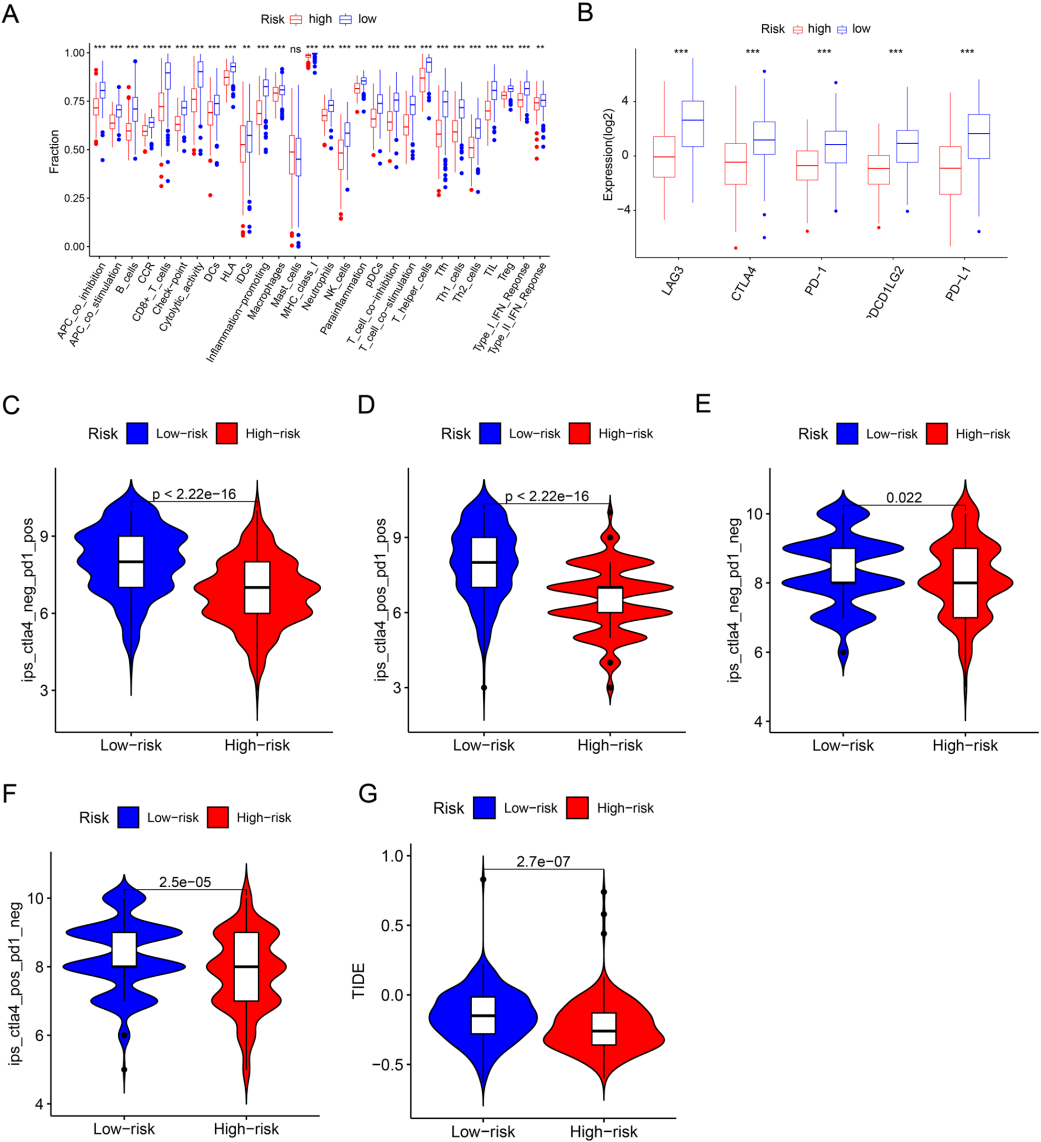
The result of immune function and immunotherapy in CM patients with different risks. (**A**) Differential results of immune function in CM patients with different risks. (**B**) Results of immune checkpoint analysis in CM patients with different risks. (**C**–**F**) Results of immunotherapy response differences in CM patients with different risks. (**G**) TIDE score of different risk patients. *p < 0.05; **p < 0.01; ***p < 0.001.

Multiple immune algorithms were used to assess the differences in immune infiltration landscape between patients in the low- and high-risk groups. Estimate results showed that patients in the low-risk group had lower tumor purity and higher stromal, immune, and estimate scores, while the opposite was true for patients in the high-risk group (Fig. 6A–D). CIBERSORT results showed that B cells naive, Plasma cells, T cells CD8, T cells CD4 memory activated , T cells follicular helper, T cells regulatory (Tregs), and Macrophages M1 were higher in the low-risk patient (Fig. 6E). In contrast, Macrophages M0, Macrophages M2, and Mast cells resting were higher in the high-risk patient population (Fig. 6E). The ssGSEA algorithm results showed that among all 23 immune cell species, except CD56 bright natural killer cellna did not differ in proportion in all patients, the other 21 immune cell were higher in the low-risk patient group than in the high-risk patient group (Fig. 6F). Correlation analysis was further performed to explore the association between prognostic related PARGs and immune infiltration landscape. The results showed a significant association between prognostic related PARGs and immune cells (Fig. 6G,H). As shown in Fig. 6G, T cells CD8, Plasma cells, T cells CD4 memory activated and T cells regulatory (Tregs) were positively correlated with ZBP1, while Macrophages M0, Macrophages M2 and NK cells activated were negatively correlated with ZBP1. Notably, for all 23 immune cells based on the ssGSEA algorithm, ZBP1 was positively correlated with most immune cells (Fig. 6H). The above results suggested significant differences in immune infiltration in CM patients, and we further evaluated patients’ immune function scores, the expression of key tumor genes, and the response to immunotherapy. The results showed that the low-risk patients exhibited higher immune function scores (Fig. 7A). The expression of key tumor genes was higher in all low-risk patients than in high-risk patients (Fig. 7B). Similarly, low-risk patients showed a better response regardless of the presence of CTAL4 or PD1 (Fig. 7C–F). And the TIDE results showed that patients in the low-risk group had higher TIDE scores, indicating a more pronounced response to immunotherapy in the low-risk group (Fig. 7G). In conclusion, these results suggest that a risk model based on the PARGs prognostic signature correlates with the immune infiltration status and immunotherapy response in CM patients.

### Drug susceptibility analysis

**Figure 8:**
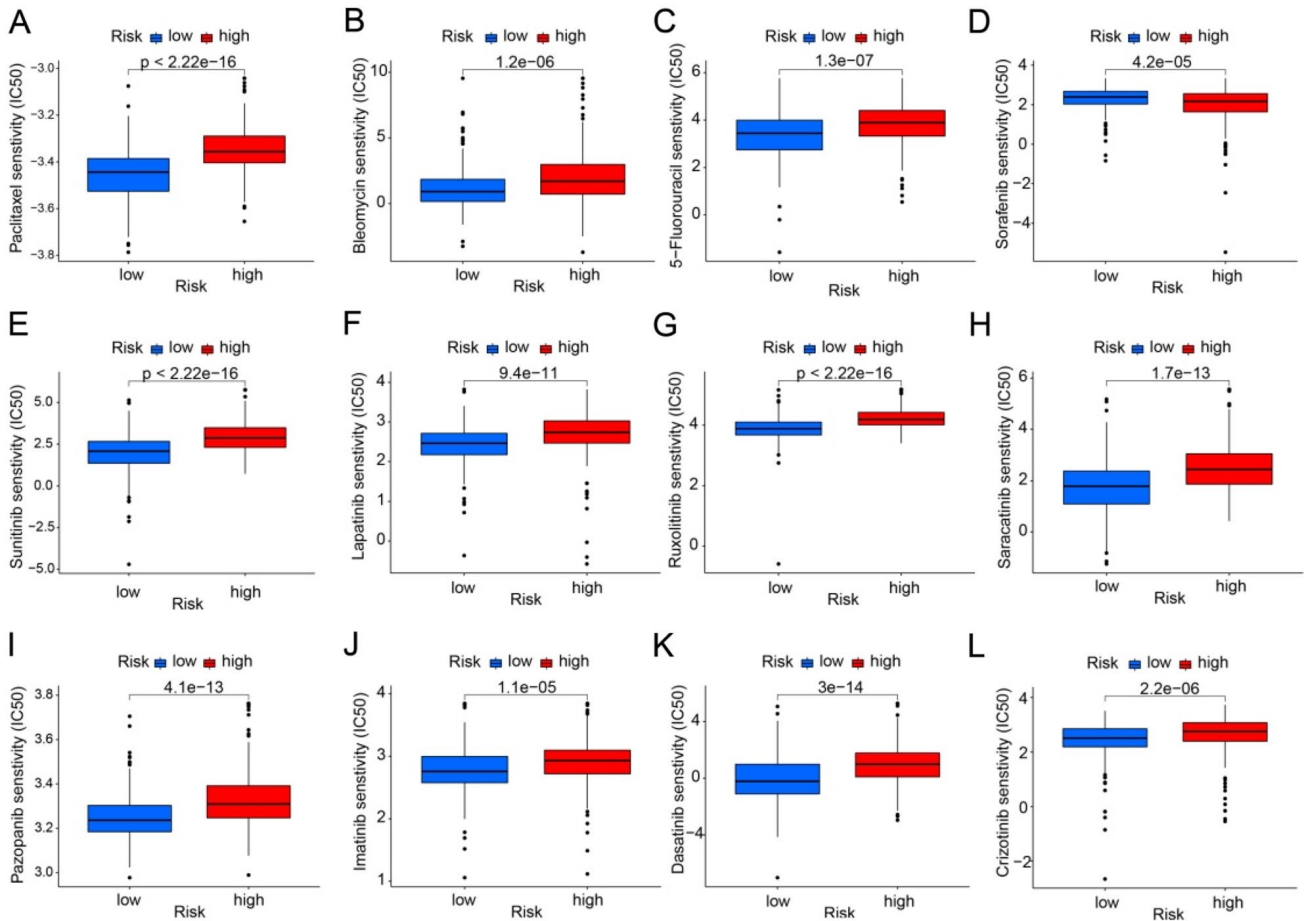
Differential analysis of drug sensitivity in CM patients with different risk groups. (**A**) Paclitaxel, (**B**) Bleomycin, (**C**) 5-Fluorouracil, (**D**) Sorafenib, (**E**) Sunitinib, (**F**) Lapatinib, (**G**) Ruxolitinib, (**H**) Saracatinib, (**I**) Pazopanib, (**J**) Imatinib, (**K**) Dasatinib and (**L**) Crizotinib. *p < 0.05; **p < 0.01; ***p < 0.001.

Antineoplastic drugs remain one of the main clinical treatments for CM. Due to the heterogeneity of CM, the sensitivity of the same drug varies for different patients, so understanding the differences in the sensitivity of antineoplastic drugs in CM patients with different risks can provide new suggestions for personalized treatment of clinical. As shown in Fig. 8, the IC50 of most antineoplastic drugs in low-risk CM patients is lower than that in high-risk CM patients, such as Paclitaxel, Dasatinib, Imatinib and Lapatinib. Notably, the IC50 of Sorafenib was higher in low-risk CM patients than in high-risk CM patients, suggesting that Sorafenib may be more effective when used in high-risk CM patients. The above results suggest that CM patients with different risks have different sensitivity to antineoplastic drugs, and the prediction of drug sensitivity in CM patients with different risks can provide new thinking for personalized treatment of patients.

### Consensus clustering analysis and immune evaluation

**Figure 9:**
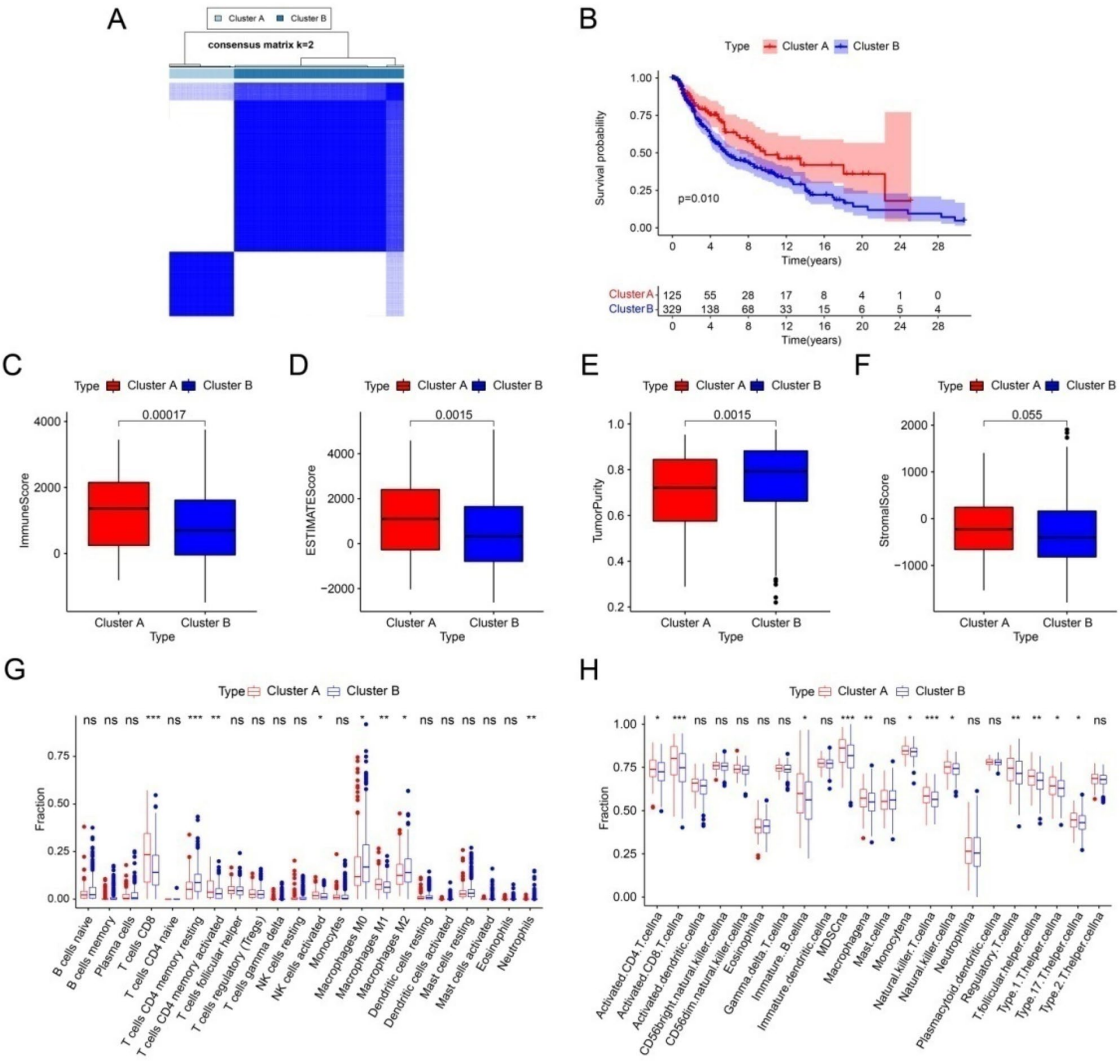
Consensus clustering of CM patients and immune landscape analysis. (**A**) Consensus clustering heat map showing the best classification of CM patients with K = 2. (**B**) The result of Kaplan–Meier survival curve in different subgroups. (**C**–**F**) Tumor purity results as well as immune, stromal and ESTIMATE scores by ESTIMATE algorithm. (**G**) Proportion of immune cells of type 22 in CM patients of different subgroups by CIBERSORT. (**H**) Proportion of immune cells of type 23 in CM patients of different subgroups by ssGSEA. *p < 0.05; **p < 0.01; ***p < 0.001.

**Figure 10:**
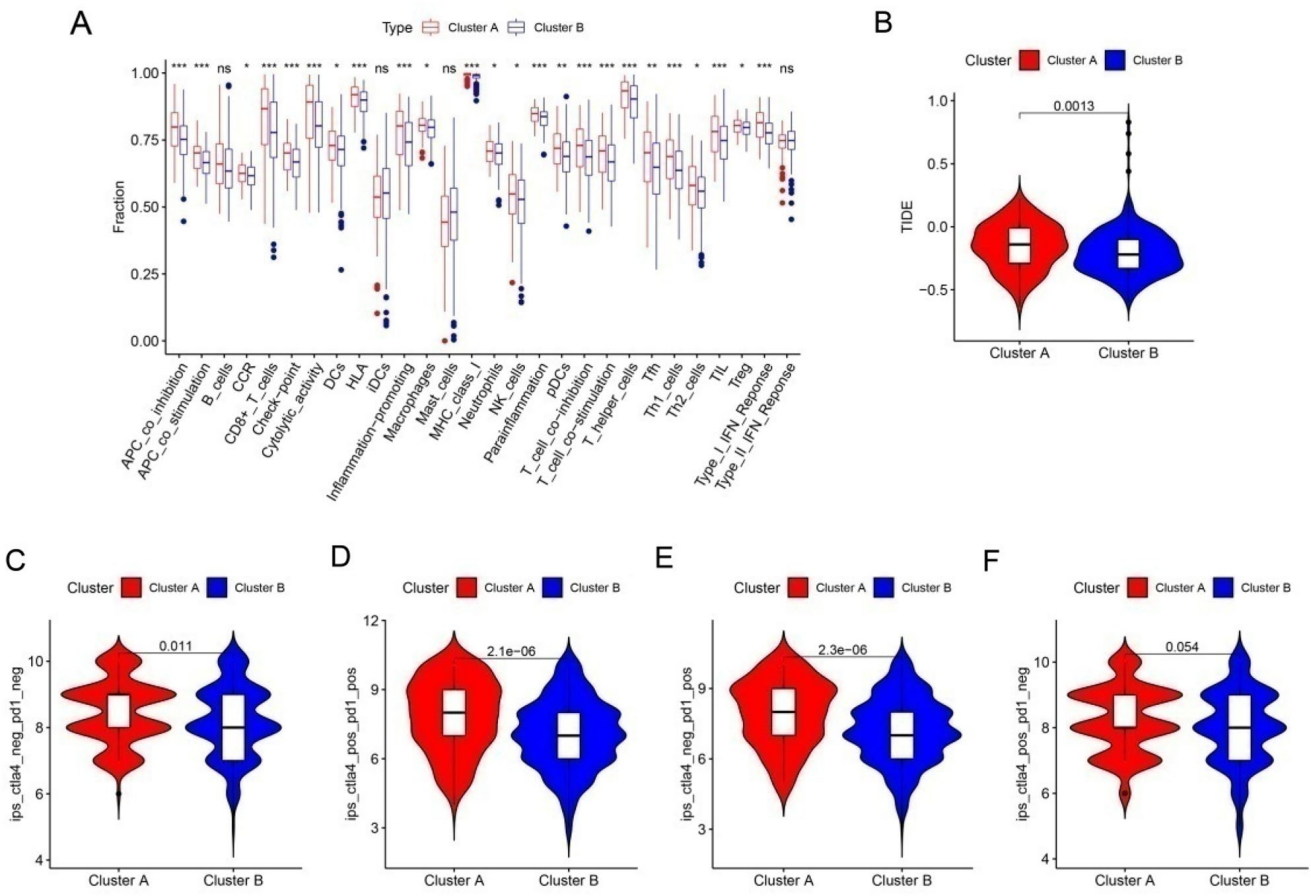
The result of immune function and immunotherapy in CM patients with different subgroups. (**A**) Differential results of immune function in CM patients with different subgroups. (**B**) TIDE score of different subgroups patients. (**C**–**F**) Results of immunotherapy response differences in CM patients with different subgroups. *p < 0.05; **p < 0.01; ***p < 0.001.

Consensus cluster analysis was used to classify CM patients into different subgroups according to the 3 prognostic related PARGs. The heat map showed that the best classification of CM patients was K = 2 with 125 samples in Cluster A and 329 samples in Cluster B (Fig. 9A). Kaplan–Meier survival analysis showed that Cluster B had a higher OS compared to Cluster A (Fig. 9B). ESTIMATE results showed that Cluster A had higher ESTIMATE, immune score, but lower tumor purity (Fig. 9C-F). Meanwhile, the CIBERSORT algorithm and ssGSEA were applied to each subgroup. As shown in Fig. 9G, T cells CD8, T cells CD4 memory activated, NK cells activated and Macrophages M1 were higher in Cluster A, while T cells CD4 memory resting, Macrophages M0 and Macrophages M2 were higher in Cluster B. The results of ssGSEA showed that the proportion of most immune cells was significantly higher in patients in Cluster A (Fig. 9H). In addition, the low-risk patients exhibited higher immune function scores (Fig. 10A). The TIDE results showed that patients in Cluster B had lower TIDE scores, which suggests that CM in Cluster B patients had a better potential immune therapy response (Fig. 10B). Similarly, low-risk patients showed a better response regardless of the presence of CTAL4 or PD1 (Fig. 10C–F). In conclusion, these results further suggest that PARGs are associated with prognosis and may indicate the immune response and immune infiltration status of CM.

## Discussion

As one of the most aggressive and metastatic malignancies, the OS of CM patients remains suboptimal^24^. PANoptosis is a recently defined inflammatory programmed cell death process involving multiple apoptotic pathways such as pyroptosis, apoptosis, and necroptosis^10–14,25,26^. A growing number of studies suggest that PANoptosis is associated with tumor development and metastasis^8,27^. However, the link between PANoptosis and CM is poorly studied, especially the exact mechanism of PARGs in CM is unclear. Therefore, we aimed to link PANoptosis to the prognosis of CM patients to reveal the role of PANoptosis and PARGs in CM. The study aims to investigate the link between PANoptosis and the prognosis of CM patients. This research could provide insights into the underlying mechanisms of CM progression and potentially lead to the development of new therapeutic strategies for CM.

In our study, ZBP1, MAP3K7 and RBCK1 were finally identified to be associated with OS in CM and could be used as reliable prognostic biomarkers for CM. A risk model was constructed based on these three prognosis-related PARGs, and the prediction accuracy of the model was better compared to other clinical characteristics. Notably, low-risk CM patients had better OS compared to high-risk CM patients. We subsequently constructed nomogram based on clinical characteristics as well as the risk model, which can be used for prediction of OS in CM patients.

The immune environment is an important factor in the interaction between tumor cells and immunity, and immune escape of tumor cells is often directly related to the effect of immunotherapy. In this study by enrichment analysis of DEGs in CM patients with different risk, we found that most immune-related pathways and biological functions were enriched, indicating that CM is very relevant to immunity. We found that the low-risk group had higher immune scores and a higher degree of immune cell infiltration by assessing the immune landscape of CM patients with different risk. The activation of the interferon signaling pathway has been shown to induce the expression of chemokines that attract T cells and NK cells, as well as the upregulation of major histocompatibility complex (MHC) molecules that present tumor antigens to T cells^28^. Therefore, the upregulation of interferon signaling pathway genes in the low-risk group may contribute to the higher infiltration of T cells CD8 and NK cells observed in these patients. In addition, the activation of the Wnt/β-catenin pathway has been associated with an immunosuppressive microenvironment characterized by the recruitment of myeloid-derived suppressor cells (MDSCs) and the inhibition of T cell activation^29^. Therefore, the upregulation of Wnt/β-catenin pathway genes in the high-risk group may contribute to the higher infiltration of Macrophages M0 and M2 observed in these patients. Notably, the prognosis-related PARGs we identified were significantly associated with immune infiltration. High expression of ZBP1, MAP3K7 and RBCK1 was accompanied by a higher degree of immune infiltration, suggesting that PANoptosis plays a key role in the immune landscape of CM.

Z-DNA binding protein 1 (ZBP1) was identified for its role in inducing tumor-associated proteins^30^. However, recent studies have shown that ZBP1 can function as a central regulator of multiple programmed death pathways^31,32^. Rajendra Karki showed that ZBP1 can mediate NLRP3 inflammatory vesicle activation and PANoptosis^33^. Yuanqin Yang found that ZBP1 can control anti-tumor immune responses through mixed-spectrum kinase structural domain-like pseudokinase (MLKL)^34^. Caspases regulate cell death, immune responses, and homeostasis, and Min Zheng found that caspase-6 can act by promoting ZBP1-mediated inflammatory vesicle activation and cell death^10^. In conjunction with our study, it is clear that ZBP1 has a very important association with immunity.

Mitogen-activated protein kinase kinase kinase 7 (MAP3K7) encodes transforming growth factor β-activated kinase 1 (TAK1) and can regulate the immune response, cell death and carcinogenesis^35^. Inokuchi S found that TAK1 deficiency can lead to cellular injury, inflammation, fibrosis and carcinogenesis^36^. More notably, TAK1 has a key role in differentiation and function of macrophage^37^. In conjunction with our study, the absence of MAP3K7 in the high-risk group may count for the worse immune environment.

Gamma interferon (IFN-γ) is a key driver of immune activation and immunosuppression^38^. RBCK1, a gene related to IFN-γ signaling, is associated with tumor immune infiltration as well as immunotherapy^39,40^. Martin Krenn found that N-terminal mutations in RBCK1 may lead to immune dysfunction^41^. RBCK1 is also able to promote NFKB signaling in the immune response through the linear ubiquitin assembly complex (LUBAC) to facilitate NFKB signaling in the immune response^42^. In our study, RBCK1 was highly associated with infiltration of many immune cells.The effect of RBCK1 on immune function may be responsible for the differences in sensitivity to immunotherapy and drug treatment in CM patients with different risks.

Overall, our study shows that PANoptosis plays a crucial role in CM. The risk models based on prognosis-related PARGs not only can effectively predict the CM patients prognosis but also indicate the immune landscape of CM patients. Understanding the role of PANoptosis and PARGs in CM could provide new avenues for targeted therapy and personalized treatment options for CM patients. This research could have a significant impact on improving the prognosis and survival rates of CM patients.

## Supplementary Information

Supplementary Information.

## Data Availability

The datasets involved in this study were obtained from public databases (TCGA, https://portal.gdc.cancer.gov/). And the raw data presented in the study are included in the article or supplementary material, further inquiries can be addressed to the respective authors.
