# Supplementary material for: PANoptosis-related prognostic signature predicts overall survival of cutaneous melanoma and provides insights into immune infiltration landscape
Source: Sci Rep. 2023 May 25;13:8449. doi: 10.1038/s41598-023-35462-4 (PMC10213036; doi:10.1038/s41598-023-35462-4)
Supplement: Supplementary file 1 — Supplementary Information. [file 41598_2023_35462_MOESM1_ESM.pdf]

Supplementary Table 1: Clinical details of the samples involved in this study

Samples	Age	Sex	Tumor tissue site	Event	T	N	M	Stage	days to last followup
**TCGA-EE-A2MK**	18	FEMALE	Extremities	Alive	T4a	N0	M0	Stage III	4403
**TCGA-EB-A85I**	66	MALE	Trunk	Alive	T4b	N0	M0	Stage IIC	-5
**TCGA-D3-A8GJ**	18	MALE	Head and Neck	Alive	T3	N0	M0	Stage II	6965
**TCGA-D3-A51J**	19	MALE	Not Available	Alive	T0	N1b	M0	Stage III	4012
**TCGA-FS-A1Z7**	19	MALE	Head and Neck	Dead	T4b	N1b	M0	Stage IIIC	Not Available
**TCGA-EE-A2MG**	23	MALE	Trunk	Dead	T2	N0	M0	Stage I	Not Available
**TCGA-D3-A2JK**	24	MALE	Trunk	Dead	T4b	N2b	M0	Stage IIIC	Not Available
**TCGA-GN-A263**	24	MALE	Trunk	Dead	T4b	N3	M1c	Stage IV	Not Available
**TCGA-WE-A8ZT**	25	FEMALE	Trunk	Alive	T3b	N1b	M1b	Stage IV	114
**TCGA-EE-A3AG**	25	MALE	Not Available	Dead	T0	N2c	M0	Stage III	714
**TCGA-D3-A8GV**	25	MALE	Extremities	Dead	TX	N0	M0	Not Available	Not Available
**TCGA-ER-A3ES**	25	MALE	Extremities	Dead	Not Available	Not Available	Not Available	Not Available	Not Available
**TCGA-EB-A4IS**	77	MALE	Trunk	Alive	T3b	NX	M0	Stage IIB	0
**TCGA-D3-A8GE**	26	MALE	Not Available	Alive	TX	N0	M1b	Stage IV	462
**TCGA-W3-AA21**	26	MALE	Extremities	Dead	T2	N0	M0	Stage I	Not Available
**TCGA-DA-A1I5**	27	FEMALE	Head and Neck	Alive	T1a	N0	M1c	Stage IV	2899
**TCGA-EB-A4XL**	56	FEMALE	Extremities	Alive	T4b	NX	M0	Stage IIC	0
**TCGA-EE-A2GS**	28	FEMALE	Extremities	Dead	T2a	N0	M0	Stage IB	1691
**TCGA-FR-A44A**	29	FEMALE	Extremities	Alive	T3a	N0	M0	Stage II	4886
**TCGA-GN-A4U3**	30	MALE	Extremities	Alive	T3a	N1a	M0	Stage III	3288
**TCGA-EE-A3AB**	30	MALE	Not Available	Alive	T0	N2a	M0	Stage III	3733
**TCGA-DA-A1IA**	32	FEMALE	Extremities	Dead	T2a	N1b	M0	Stage IIIB	1887
**TCGA-FS-A1Z0**	32	FEMALE	Extremities	Dead	T1a	N0	M0	Stage IA	Not Available
**TCGA-EB-A431**	34	MALE	Extremities	Alive	T4b	N0	M0	Stage IIC	52
**TCGA-FW-A3TV**	57	FEMALE	Trunk	Alive	T1	N2b	M0	Stage IIIB	5
**TCGA-FR-A69P**	34	FEMALE	Not Available	Alive	TX	N3		Stage IIIC	149
**TCGA-EB-A550**	75	FEMALE	Unknown	Dead	T4b	NX	M0	Stage IIC	6
**TCGA-FW-A3I3**	59	FEMALE	Not Available	Alive		N0	M1	Stage IV	6
**TCGA-EB-A5FP**	65	FEMALE	Trunk	Dead	T4b	NX	M1b	Stage IV	6
**TCGA-EE-A2GH**	34	MALE	Trunk	Alive	T2	N0	M0	Stage I	5536
**TCGA-EE-A2MP**	34	FEMALE	Extremities	Alive	T2	N0	M0	Stage I	7563
**TCGA-EE-A2ML**	35	MALE	Trunk	Dead	T3a	N0	M0	Stage II	6176

**TCGA-ER-A19L**	35	MALE	Extremities	Dead	Not Available	Not Available	Not Available	Not Available	Not Available
**TCGA-EB-A551**	78	FEMALE	Extremities	Alive	T4b	N2b	M0	Stage IIIC	8
**TCGA-EB-A3XC**	74	MALE	Trunk	Alive	T4b	N0	M0	Stage IIC	9
**TCGA-ER-A19M**	36	MALE	Extremities	Dead	T2a	N0	M0	Stage IB	Not Available
**TCGA-BF-AAOX**	83	MALE	Extremities	Alive	T4b	N0	M0	Stage IIC	11
**TCGA-DA-A1HW**	37	FEMALE	Trunk	Dead	T1a	N1b	M0	Stage IIIB	59
**TCGA-WE-AA9Y**	37	MALE	Extremities	Alive	T2a	N3	M0	Stage IIIC	67
**TCGA-D3-A2JP**	37	MALE	Not Available	Alive	T0	N3	M0	Stage IIIC	1372
**TCGA-BF-A1PZ**	71	FEMALE	Extremities	Alive	T4a	N0	M0	Stage IIB	12
**TCGA-BF-A9VF**	77	MALE	Extremities	Alive	T4b	N0	M0	Stage IIC	12
**TCGA-FR-A729**	38	FEMALE	Trunk	Alive	T1	N0	M0	Stage I	6390
**TCGA-GN-A267**	38	MALE	Trunk	Dead	T4a	N1a	M0	Stage IIIA	Not Available
**TCGA-D9-A3Z3**	39	FEMALE	Unknown	Alive	T3a	N1b	M0	Stage IIIB	99
**TCGA-DA-A3F8**	39	MALE	Head and Neck	Alive	T2a	N2b	M0	Stage IIIB	111
**TCGA-EE-A2GI**	39	MALE	Extremities	Alive	T1a	N0	M0	Stage IA	686
**TCGA-D3-A3CB**	39	MALE	Trunk	Alive	T2	N0	M0	Not Available	4511
**TCGA-EB-A553**	62	MALE	Trunk	Alive	T4b	N0	M0	Stage IIC	13
**TCGA-XV-AAZY**	76	FEMALE	Trunk	Alive	T4	N3	M0	Stage IIIC	13
**TCGA-BF-A3DM**	63	MALE	Trunk	Alive	T2b	N0	M0	Stage IIA	14
**TCGA-ER-A2NE**	39	MALE	Extremities	Dead	Tis	N0	M0	Stage 0	Not Available
**TCGA-FS-A1YX**	39	FEMALE	Trunk	Dead	T2	N0	M0	Stage I	Not Available
**TCGA-BF-AAP0**	40	FEMALE	Trunk	Alive	T4	NX	M1	Stage IV	14
**TCGA-FS-A1ZE**	40	MALE	Trunk	Dead	T4b	N0	M0	Stage IIC	1225
**TCGA-EE-A2GL**	40	FEMALE	Extremities	Alive	T3a	N0	M0	Stage IIA	1673
**TCGA-D9-A148**	40	MALE	Trunk	Alive	TX	N3	M1b	Not Available	4511
**TCGA-EB-A4OZ**	41	FEMALE	Trunk	Alive	T4a	N3	M0	Stage IIIC	-3
**TCGA-BF-AAP4**	61	MALE	Trunk	Alive	T4b	N0	M0	Stage IIC	15
**TCGA-EB-A4IQ**	42	FEMALE	Trunk	Dead	T4b	N1	M0	Stage IIIB	4
**TCGA-D3-A3MR**	42	MALE	Not Available	Alive	T0	N1b	M0	Stage III	2724
**TCGA-EB-A3Y6**	56	FEMALE	Extremities	Alive	T4b	N0	M0	Stage IIC	16
**TCGA-EE-A3J3**	42	MALE	Trunk	Dead	T2	N0	M0	Stage IB	Not Available
**TCGA-ER-A19B**	42	MALE	Trunk	Dead	TX	N0	M0	Not Available	Not Available
**TCGA-D3-A5GT**	43	MALE	Trunk	Alive	T2b	N3	M0	Stage IIIC	179
**TCGA-BF-A1Q0**	80	MALE	Trunk	Alive	T4b	N0	M0	Stage IIC	17
**TCGA-YD-A89C**	43	FEMALE	Extremities	Alive	T1a	NX	M0	Stage IA	210

**TCGA-ER-A2NG**	43	FEMALE	Trunk	Dead	T3b	N3	M0	Stage IIIC	951
**TCGA-EE-A3J7**	43	MALE	Trunk	Alive	T2	N0	M0	Stage I	1949
**TCGA-EE-A2A5**	43	MALE	Extremities	Dead	T2a	N0	M0	Stage IB	Not Available
**TCGA-DA-A1HV**	75	FEMALE	Not Available	Alive	T0	N2b	M0	Stage IIIB	26
**TCGA-EE-A2MI**	43	MALE	Extremities	Dead	T4	N0	M0	Stage IIB	Not Available
**TCGA-FS-A1ZN**	43	MALE	Trunk	Dead	T4b	N1a	M0	Stage IIIA	Not Available
**TCGA-D3-A8GL**	43	MALE	Extremities	Dead	T2a	N1b	M0	Stage IIIB	Not Available
**TCGA-FR-A3YN**	44	MALE	Extremities	Alive	T2a	N0	M0	Stage IB	2449
**TCGA-EE-A2GE**	44	MALE	Trunk	Alive	T2	N0	M0	Stage I	4135
**TCGA-EE-A3JA**	44	MALE	Extremities	Dead	T2a	N0	M0	Stage IB	Not Available
**TCGA-EB-A3XB**	63	MALE	Trunk	Alive	T4	NX	M0	Stage II	36
**TCGA-EB-A3HV**	37	MALE	Trunk	Alive	T4b	N0	M0	Stage IIC	39
**TCGA-EB-A24D**	72	MALE	Extremities	Alive	T4a	N2b	M0	Stage IIIB	42
**TCGA-EB-A44N**	59	MALE	Extremities	Dead	T4b	N0	M0	Stage IIC	45
**TCGA-EE-A2MT**	45	MALE	Trunk	Alive	T2a	N0	M0	Stage IB	1265
**TCGA-DA-A1I2**	45	MALE	Trunk	Dead	T4b	N2b	M0	Stage III	4719
**TCGA-FW-A5DX**	71	MALE	Trunk	Alive	T4a	N3	Not Available	Stage IIIC	59
**TCGA-D3-A8GK**	45	MALE	Extremities	Alive	T3a	N0	M0	Stage IIA	4757
**TCGA-DA-A3F5**	45	MALE	Trunk	Dead	T1a	N0	M0	Stage I	6076
**TCGA-FS-A1ZA**	45	FEMALE	Trunk	Dead	T4b	N2c	M0	Stage IIIB	Not Available
**TCGA-ER-A198**	45	MALE	Other Specify	Dead	Not Available	NX	M0	Not Available	Not Available
**TCGA-D9-A1JW**	82	MALE	Head and Neck	Alive	T1a	N2a	M0	Not Available	82
**TCGA-GN-A266**	45	MALE	Not Available	Dead	Not Available	Not Available	Not Available	Not Available	Not Available
**TCGA-BF-A1PU**	46	FEMALE	Extremities	Alive	T4b	N0	M0	Stage IIC	387
**TCGA-EE-A2GK**	46	FEMALE	Trunk	Alive	T1	N0	M0	Stage I	1665
**TCGA-D3-A2JN**	46	FEMALE	Not Available	Dead	T0	N1b	M0	Stage III	1709
**TCGA-EE-A2A1**	46	MALE	Extremities	Alive	T2a	N0	M0	Stage IB	2402
**TCGA-ER-A19D**	46	FEMALE	Trunk	Dead	T2a	N0	M0	Stage IB	Not Available
**TCGA-FS-A4F2**	46	FEMALE	Head and Neck	Dead	T4b	N0	M0	Stage IIC	Not Available
**TCGA-FS-A4FB**	46	FEMALE	Extremities	Dead	T2	N1a	M0	Stage III	Not Available
**TCGA-HR-A2OH**	46	FEMALE	Extremities	Dead	T3b	N2a	M0	Stage IIIB	Not

										Available
**TCGA-WE-AAA0**	47	MALE	Head and Neck	Alive	T1a	N0	M0	Stage IA	918
**TCGA-GN-A262**	47	FEMALE	Trunk	Alive	Not Available	Not Available	Not Available	Not Available	3664
**TCGA-EE-A3AA**	47	MALE	Not Available	Alive	T0	N2a	M0	Stage III	3781
**TCGA-EB-A5VV**	74	FEMALE	Head and Neck	Alive	T3b	N1	M0	Stage IIIB	193
**TCGA-ER-A19N**	47	MALE	Not Available	Dead	Not Available	Not Available	Not Available	Not Available	Not Available
**TCGA-EB-A5UM**	48	FEMALE	Trunk	Alive	T4b	N0	M0	Stage IIC	0
**TCGA-QB-A6FS**	49	MALE	Not Available	Alive	T0	N3	M0	Stage IIIC	203
**TCGA-ER-A42K**	40	FEMALE	Extremities	Dead	T4b	N3	M0	Stage IIIC	206
**TCGA-GF-A2C7**	48	MALE	Head and Neck	Alive	T4b	N0	M0	Stage IIC	1
**TCGA-EB-A1NK**	48	MALE	Extremities	Alive	T4b	N0	M0	Stage IIC	16
**TCGA-DA-A1I0**	63	MALE	Trunk	Dead	T4b	N3	M1a	Stage IV	215
**TCGA-GF-A4EO**	74	FEMALE	Not Available	Alive	T0	N3	M0	Stage IIIC	220
**TCGA-FW-A5DY**	48	FEMALE	Trunk	Alive	T3	N1	Not Available	Stage III	73
**TCGA-D9-A1X3**	63	MALE	Extremities	Alive	T4b	N2b	Not Available	Not Available	237
**TCGA-EE-A29R**	48	FEMALE	Head and Neck	Alive	T3b	N1b	M0	Stage IIIC	440
**TCGA-ER-A19G**	48	FEMALE	Extremities	Alive	Not Available	N0	M0	Not Available	8180
**TCGA-GN-A4U7**	56	FEMALE	Extremities	Dead	T2b	N3	M0	Stage IIIC	266
**TCGA-DA-A3F2**	55	MALE	Trunk	Dead	T4a	N2b	M0	Stage IIIB	287
**TCGA-EE-A183**	48	MALE	Trunk	Dead	Tis	N0	M0	Stage 0	Not Available
**TCGA-D3-A2J8**	48	MALE	Extremities	Dead	T2a	N0	M0	Stage IB	Not Available
**TCGA-D3-A8GB**	48	MALE	Extremities	Dead	T3a	N1b	M0	Stage IIIB	Not Available
**TCGA-EE-A3AF**	48	FEMALE	Not Available	Dead	T0	N3	M0	Stage IIIC	Not Available
**TCGA-ER-A19W**	48	FEMALE	Trunk	Dead	Not Available	Not Available	Not Available	Not Available	Not Available
**TCGA-EB-A44R**	52	MALE	Extremities	Dead	TX	N2b	M0	Stage IIIB	309
**TCGA-WE-AAA3**	84	FEMALE	Extremities	Alive	T4b	N2b	M0	Stage IIIC	315
**TCGA-EB-A42Z**	49	MALE	Extremities	Alive	T4b	N1b	M0	Stage IIIC	12
**TCGA-WE-A8ZR**	49	MALE	Trunk	Dead	T4b	N1b	M0	Stage IIIC	133
**TCGA-D9-A3Z1**	66	MALE	Unknown	Dead	T2a	N3	M0	Stage IIIC	345
**TCGA-D3-A8GD**	63	FEMALE	Extremities	Alive	T4b	N3	M0	Stage IIIC	353
**TCGA-Z2-AA3V**	57	FEMALE	Trunk	Alive	T1a	N0	M0	Stage IA	357
**TCGA-ER-A2NH**	49	MALE	Trunk	Alive	T3a	N3	M0	Stage IIIC	975
**TCGA-EB-A6L9**	55	MALE	Extremities	Alive	TX	N3	M0	Stage IIIC	371
**TCGA-ER-A42L**	49	MALE	Other Specify	Alive	T3	N0	M0	Stage II	3812

**TCGA-OD-A75X**	49	MALE	Trunk	Dead	TX	NX	M0	Not Available	8966
**TCGA-EE-A2M5**	49	MALE	Trunk	Dead	T2	N0	M0	Stage I	Not Available
**TCGA-FR-A2OS**	49	FEMALE	Extremities	Dead	T4b	N0	M0	Stage IIC	Not Available
**TCGA-HR-A2OG**	50	FEMALE	Extremities	Alive	Not Available	Not Available	Not Available	Not Available	7
**TCGA-D9-A4Z2**	50	MALE	Trunk;Extremities	Dead	T4b	N3	M0	Stage IIIC	93
**TCGA-YG-AA3P**	63	FEMALE	Extremities	Alive	T4a	N0	M0	Stage IIB	439
**TCGA-EE-A3AD**	50	MALE	Not Available	Dead	T0	N1b	M0	Stage III	112
**TCGA-D3-A2JO**	50	FEMALE	Extremities	Alive	TX	N3	M0	Stage IIIC	1548
**TCGA-ER-A2NC**	50	MALE	Extremities	Dead	T2a	N0	M0	Stage IB	Not Available
**TCGA-FR-A7U8**	50	MALE	Not Available	Alive	TX	N3	M0	Stage IIIC	461
**TCGA-XV-A9W5**	51	MALE	Extremities	Alive	T2	N0	M0	Not Available	15
**TCGA-BF-A3DJ**	36	FEMALE	Extremities	Alive	T4b	N1	M0	Stage IIIB	464
**TCGA-EB-A44Q**	51	FEMALE	Extremities	Alive	TX	N3	M0	Stage IIIC	28
**TCGA-ER-A2NB**	57	MALE	Other Specify	Dead	T4b	N2	M0	Stage IIIB	486
**TCGA-D3-A51K**	51	MALE	Extremities	Alive	Tis	N2b	M0	Stage IIIB	559
**TCGA-IH-A3EA**	61	MALE	Head and Neck	Alive	T4b	N0	M0	Stage IIC	524
**TCGA-DA-A1I4**	51	MALE	Trunk	Dead	T3b	N2b	M0	Stage IIIC	823
**TCGA-D3-A51F**	51	MALE	Extremities	Alive	T4b	N1b	M0	Stage IIIC	1329
**TCGA-EE-A2GT**	77	MALE	Trunk	Alive	T3a	N0	M0	Stage IIA	595
**TCGA-D3-A3MV**	38	FEMALE	Extremities	Alive	T2b	N2a	M0	Stage IIIB	653
**TCGA-GN-A4U8**	51	MALE	Trunk	Alive	Not Available	Not Available	Not Available	Not Available	1410
**TCGA-WE-A8ZX**	45	MALE	Not Available	Alive	TX	N1b	M0	Stage IIIB	690
**TCGA-FW-A3R5**	68	MALE	Head and Neck	Alive	TX	N2	M0	Stage III	697
**TCGA-GN-A4U5**	61	FEMALE	Trunk	Alive	T2a	NX	M0	Stage IB	707
**TCGA-EE-A2GB**	51	MALE	Trunk	Alive	T2b	N1a	M0	Stage IIIB	1446
**TCGA-Z2-A8RT**	42	FEMALE	Extremities	Alive	T3b	N0	M0	Stage IIB	719
**TCGA-EE-A2ME**	51	MALE	Trunk	Dead	T2	N0	M0	Stage I	Not Available
**TCGA-ER-A19T**	51	MALE	Extremities	Dead	T4a	N3	M1a	Stage IV	Not Available
**TCGA-FS-A1ZC**	51	MALE	Trunk	Dead	TX	N0	M0	Not Available	Not Available
**TCGA-GN-A4U4**	73	MALE	Trunk	Alive	T2b	NX	M0	Stage IIA	784
**TCGA-EE-A3AC**	47	MALE	Not Available	Alive	T0	N2b	M0	Stage III	786
**TCGA-DA-A95W**	52	MALE	Not Available	Alive	TX	N1b	M0	Stage IIIB	31
**TCGA-DA-A3F3**	52	MALE	Not Available	Dead	T0	N2b	M0	Stage IIIB	151
**TCGA-D3-A8GS**	52	MALE	Trunk	Dead	T1	N0	M0	Stage I	Not

										Available
**TCGA-FS-A1YW**	52	MALE	Trunk	Dead	T1b	N0	M0	Stage IB	Not Available
**TCGA-FS-A1ZP**	52	MALE	Extremities	Dead	T3	N0	M0	Stage II	Not Available
**TCGA-EE-A2MD**	52	MALE	Extremities	Dead	T3a	N0	M0	Stage II	Not Available
**TCGA-EB-A51B**	53	MALE	Trunk	Alive	T4b	NX	M0	Stage IIC	-5
**TCGA-EB-A3XD**	53	FEMALE	Extremities	Alive	T4b	NX	M0	Stage IIC	0
**TCGA-EE-A29H**	59	FEMALE	Trunk	Alive	T1a	N0	M0	Stage IA	979
**TCGA-ER-A2NF**	53	MALE	Head and Neck	Dead	T3b	N3	M0	Stage IIIB	498
**TCGA-D3-A3C7**	57	FEMALE	Not Available	Alive	T0	N1b	M0	Stage III	1007
**TCGA-ER-A3PL**	30	MALE	Extremities	Alive	T3b	N0	M1a	Stage IV	1010
**TCGA-D3-A3MU**	53	MALE	Trunk	Alive	T3a	N2a	M0	Stage IIIA	819
**TCGA-D3-A3C8**	58	FEMALE	Head and Neck	Alive	TX	N3	M0	Stage IIIC	1038
**TCGA-D3-A2JC**	53	FEMALE	Not Available	Alive	T0	N2b	M0	Stage III	2126
**TCGA-EE-A20F**	53	MALE	Extremities	Alive	T1	N0	M0	Stage I	2399
**TCGA-GN-A265**	53	MALE	Not Available	Alive	Not Available	Not Available	Not Available	Not Available	2577
**TCGA-EE-A29G**	53	MALE	Head and Neck	Dead	T4a	N2a	M0	Stage IIIA	Not Available
**TCGA-XV-AB01**	54	FEMALE	Head and Neck	Alive	T3	NX	M0	Stage II	19
**TCGA-DA-A95X**	62	MALE	Extremities	Alive	T2a	N0	M0	Stage IB	1144
**TCGA-D9-A3Z4**	54	MALE	Unknown	Dead	T4b	N3	M0	Stage IIIC	119
**TCGA-EB-A5UN**	49	MALE	Trunk	Alive	T4b	NX	M0	Stage IIC	1182
**TCGA-FR-A728**	54	FEMALE	Extremities	Alive	T4b	N2a	M0	Stage IIIB	247
**TCGA-EE-A29E**	54	MALE	Extremities	Alive	T3a	N1b	M0	Stage IIIB	1205
**TCGA-D3-A51H**	60	MALE	Trunk	Alive	T1b	N3	M0	Stage IIIC	1221
**TCGA-D9-A4Z6**	54	MALE	Trunk	Dead	T3b	N1b	M0	Stage IIIC	338
**TCGA-FS-A1ZS**	54	MALE	Trunk	Alive	T2	N0	M0	Stage I	4526
**TCGA-EE-A2GC**	82	MALE	Head and Neck	Alive	T3b	N0	M0	Stage IIB	1316
**TCGA-D3-A3C6**	54	FEMALE	Extremities	Dead	T2a	N0	M0	Stage IB	Not Available
**TCGA-WE-A8ZY**	62	MALE	Trunk	Dead	T3a	NX	M0	Stage IIA	1330
**TCGA-EE-A29M**	33	FEMALE	Extremities	Alive	T2a	N0	M0	Stage IB	1349
**TCGA-FS-A1ZZ**	54	FEMALE	Trunk	Dead	T3b	N0	M0	Stage IIB	Not Available
**TCGA-EB-A5SG**	57	FEMALE	Extremities	Alive	Not Available	Not Available	Not Available	Not Available	1372
**TCGA-EE-A2M8**	54	FEMALE	Extremities	Dead	T3a	N1	M0	Stage III	Not Available
**TCGA-BF-AAP6**	55	MALE	Extremities	Alive	T4b	N2	M0	Stage III	15
**TCGA-FW-A3TU**	72	FEMALE	Trunk	Dead	Not Available	Not Available	Not Available	Not Available	1446

**TCGA-EE-A29C**	20	MALE	Trunk	Dead	T2a	N0	M0	Stage IB	1455
**TCGA-EB-A5KH**	55	MALE	Extremities	Dead	T0	N1	M0	Stage III	543
**TCGA-D3-A2JF**	74	MALE	Extremities;Trunk	Alive	T1a	N0	M0	Stage IA	1483
**TCGA-DA-A1I7**	62	MALE	Not Available	Alive	T0	N2b	M0	Stage IIIB	1495
**TCGA-D3-A1QA**	55	MALE	Extremities	Alive	T2a	N0	M0	Stage IB	2326
**TCGA-DA-A1I1**	55	MALE	Not Available	Alive	T0	N2a	M0	Stage III	4830
**TCGA-FS-A1YY**	55	FEMALE	Trunk	Dead	T3a	N0	M0	Stage IIA	Not Available
**TCGA-ER-A3EV**	55	MALE	Extremities	Dead	T4	N0	M0	Stage III	Not Available
**TCGA-D3-A1Q6**	55	MALE	Extremities	Dead	T4	N1b	M0	Stage III	Not Available
**TCGA-EE-A185**	55	FEMALE	Extremities	Dead	T4b	N3	M0	Stage IIIC	Not Available
**TCGA-WE-A8ZQ**	48	MALE	Extremities	Alive	T3a	N0	M0	Stage IIA	1592
**TCGA-FS-A1ZT**	55	MALE	Extremities	Alive	T2	N1b	M0	Stage III	1617
**TCGA-EE-A2MU**	71	MALE	Extremities	Alive	T1a	N0	M0	Stage IA	1620
**TCGA-EB-A57M**	56	MALE	Extremities	Dead	T4b	N1	M0	Stage IIIB	8
**TCGA-EB-A5VU**	56	MALE	Extremities	Dead	T4b	N1	M0	Stage IIIB	12
**TCGA-EB-A24C**	56	MALE	Trunk	Alive	T4b	NX	M0	Not Available	14
**TCGA-XV-AAZV**	56	FEMALE	Trunk	Alive	T4	N0	M0	Stage II	17
**TCGA-EE-A29S**	79	MALE	Extremities	Dead	T3a	N0	M0	Stage IIA	1701
**TCGA-D3-A51N**	56	FEMALE	Not Available	Alive	T0	N3	M1c	Stage IV	305
**TCGA-EE-A29P**	73	FEMALE	Trunk	Alive	T4b	N0	M0	Stage IIC	1716
**TCGA-EE-A2A6**	43	MALE	Trunk	Alive	T1a	N0	M0	Stage IA	1787
**TCGA-WE-A8ZO**	73	FEMALE	Extremities	Alive	T3a	N1b	M0	Stage IIIB	1807
**TCGA-WE-AAA4**	56	FEMALE	Extremities	Alive	TX	N3	M0	Stage IIIC	461
**TCGA-FR-A8YD**	56	FEMALE	Other Specify	Dead	T4b	N0	M0	Stage IIC	896
**TCGA-D9-A6EC**	56	MALE	Trunk	Alive	T3a	N1	M0	Stage IIIA	2015
**TCGA-EE-A20H**	56	MALE	Extremities	Dead	T2	N0	M0	Stage I	Not Available
**TCGA-BF-A1PX**	56	MALE	Trunk	Dead	T4b	N2a	M0	Stage IIIB	Not Available
**TCGA-EB-A3XF**	57	MALE	Trunk	Alive	T4b	N0	M0	Stage IIC	4
**TCGA-WE-A8ZN**	57	MALE	Extremities	Alive	T4a	NX	M0	Stage IIB	1463
**TCGA-FS-A1ZB**	57	MALE	Trunk	Dead	T3a	N0	M0	Stage II	Not Available
**TCGA-EE-A17Z**	57	MALE	Trunk	Dead	T4a	N0	M0	Stage IIB	Not Available
**TCGA-W3-AA1Q**	57	MALE	Not Available	Dead	TX	N1	M0	Stage III	Not Available
**TCGA-ER-A2ND**	57	FEMALE	Extremities	Dead	T1b	N3	M0	Stage IIIC	Not Available

**TCGA-EB-A6R0**	58	FEMALE	Trunk	Dead	T4b	N0	M0	Stage IIC	-2
**TCGA-EB-A44P**	58	FEMALE	Trunk	Alive	T4b	N0	M0	Stage IIC	7
**TCGA-FS-A4FD**	39	MALE	Trunk;Not Available	Dead	T2	N3	M0	Stage IIIC	2369
**TCGA-BF-AAP8**	58	MALE	Extremities	Alive	T4b	N0	M0	Stage IIC	14
**TCGA-EB-A82B**	58	FEMALE	Extremities	Alive	T4b	N2	M0	Stage III	14
**TCGA-D3-A5GS**	58	MALE	Trunk	Alive	T1b	N1b	M1c	Stage IV	226
**TCGA-GF-A3OT**	58	FEMALE	Extremities	Alive	T3	N3	M0	Stage IIIC	301
**TCGA-ER-A1A1**	58	MALE	Not Available	Alive	TX	N3	M0	Stage IIIC	2433
**TCGA-EE-A29B**	67	MALE	Extremities	Dead	T3b	N0	M0	Stage IIB	2452
**TCGA-Z2-AA3S**	58	MALE	Trunk	Alive	T1a	N0	M0	Stage IA	2831
**TCGA-EE-A2GD**	58	FEMALE	Extremities	Dead	T4	N0	M0	Stage IIB	9568
**TCGA-EE-A2MN**	58	MALE	Trunk	Dead	T2	N0	M0	Stage I	Not Available
**TCGA-EE-A29X**	58	FEMALE	Head and Neck	Dead	T2a	N0	M0	Stage IB	Not Available
**TCGA-D3-A51T**	59	FEMALE	Trunk	Alive	T4b	N1b	M0	Stage IIIC	458
**TCGA-EE-A20C**	59	MALE	Extremities	Dead	Tis	N0	M0	Stage 0	Not Available
**TCGA-FR-A8YE**	41	MALE	Trunk	Alive	T1a	N0	M0	Stage IA	2871
**TCGA-D3-A1Q4**	53	FEMALE	Extremities	Alive	T2b	N1b	M0	Stage IIIC	2885
**TCGA-GN-A9SD**	59	FEMALE	Extremities	Dead	T1a	NX	M0	Stage IA	Not Available
**TCGA-EE-A3J8**	59	MALE	Trunk	Dead	T4a	N1a	M0	Stage IIIA	Not Available
**TCGA-EB-A5SH**	60	FEMALE	Extremities	Alive	T4	N0	M0	Stage III	1065
**TCGA-EE-A3JH**	54	MALE	Trunk	Alive	T2	N0	M0	Stage IB	3096
**TCGA-D3-A51R**	60	MALE	Extremities	Alive	T3a	N0	M0	Stage IIA	1572
**TCGA-D3-A2JA**	68	MALE	Trunk	Alive	T2a	N1a	M0	Stage IIIA	3104
**TCGA-EE-A2MR**	61	MALE	Trunk	Alive	T2	N0	M0	Stage I	3150
**TCGA-DA-A1HY**	42	MALE	Extremities	Alive	T2b	N1	M0	Stage III	3199
**TCGA-EE-A3JB**	60	FEMALE	Extremities	Alive	T3a	N1	M0	Stage III	6138
**TCGA-W3-A825**	60	FEMALE	Extremities	Dead	T3	N0	M0	Stage II	Not Available
**TCGA-D3-A5GL**	74	MALE	Extremities	Alive	T2a	N0	M0	Stage IB	3374
**TCGA-EE-A2MJ**	60	MALE	Trunk	Dead	T4b	N0	M0	Stage III	Not Available
**TCGA-FS-A1ZG**	60	FEMALE	Extremities	Dead	T4b	N2b	M0	Stage IIIC	Not Available
**TCGA-D3-A1Q7**	42	FEMALE	Trunk	Alive	T1b	N0	M0	Stage IB	3687
**TCGA-D3-A1Q5**	60	MALE	Trunk	Dead	TX	N0	M0	Not Available	Not Available
**TCGA-GN-A264**	60	MALE	Extremities	Dead	Not Available	Not Available	Not Available	Not Available	Not Available
**TCGA-D3-A5GU**	36	MALE	Extremities	Alive	T1b	N0	M0	Stage IB	3808

**TCGA-EE-A2M6**	61	MALE	Trunk	Alive	T1	N0	M0	Stage I	3932
**TCGA-D3-A3CF**	61	FEMALE	Other Specify	Dead	T4b	N3	M0	Stage IIIC	Not Available
**TCGA-BF-AAP2**	62	MALE	Extremities	Alive	T3b	N0	M0	Stage IIB	13
**TCGA-D3-A5GN**	15	FEMALE	Trunk	Alive	T1	N0	M0	Stage I	4129
**TCGA-XV-AAZW**	62	FEMALE	Extremities	Dead	T4	N0	M0	Stage II	18
**TCGA-GF-A6C8**	62	FEMALE	Extremities	Alive	T3b	NX	M0	Stage IIB	62
**TCGA-YG-AA3O**	62	MALE	Trunk	Dead	Not Available	Not Available	Not Available	Not Available	1096
**TCGA-D3-A5GO**	61	MALE	Head and Neck	Alive	T4	N0	M0	Stage II	4195
**TCGA-EB-A299**	63	MALE	Extremities	Alive	T2b	N0	M0	Stage IIA	0
**TCGA-EE-A3JI**	48	MALE	Extremities;Trunk	Dead	T2	N0	M0	Stage I	4504
**TCGA-BF-A5ER**	63	MALE	Trunk	Alive	T4b	N0	M0	Stage IIC	12
**TCGA-BF-A5EQ**	63	MALE	Trunk	Alive	T4b	N0	M0	Stage IIC	12
**TCGA-FR-A7U9**	63	FEMALE	Extremities	Alive	T3b	N3	M0	Stage IIIC	213
**TCGA-D3-A8GN**	27	FEMALE	Not Available	Alive	TX	N0	M0	Not Available	4529
**TCGA-DA-A1I8**	63	FEMALE	Extremities	Dead	T4b	N0	M0	Stage IIC	999
**TCGA-D3-A3BZ**	63	MALE	Head and Neck	Alive	T4a	N0	M0	Stage IIB	3516
**TCGA-D3-A2JL**	43	FEMALE	Extremities	Alive	TX	N0	M0	Not Available	4757
**TCGA-W3-A824**	63	MALE	Trunk	Alive	T2	N0	M0	Stage I	6455
**TCGA-FS-A1ZD**	63	MALE	Extremities	Dead	T2b	N0	M0	Stage IIA	Not Available
**TCGA-D3-A51E**	39	FEMALE	Extremities	Alive	T2	N0	M0	Not Available	4923
**TCGA-D3-A5GR**	23	FEMALE	Extremities	Alive	T1b	N1	M0	Stage III	5055
**TCGA-EE-A29W**	42	MALE	Extremities	Alive	Tis	N0	M0	Stage 0	5218
**TCGA-GN-A26A**	63	FEMALE	Trunk	Dead	T3a	N1a	M0	Stage IIIA	Not Available
**TCGA-ER-A196**	64	FEMALE	Extremities	Alive	T4b	N0	M0	Stage IIC	477
**TCGA-ER-A3ET**	64	FEMALE	Other Specify	Dead	T3a	N1a	M0	Stage IIIA	2443
**TCGA-W3-AA1W**	64	MALE	Trunk	Alive	T3	N0	M0	Stage II	6301
**TCGA-FS-A4F4**	64	MALE	Extremities	Dead	T3a	N0	M0	Stage II	Not Available
**TCGA-EB-A4OY**	65	FEMALE	Extremities	Alive	T4b	N1a	M0	Stage IIIB	-2
**TCGA-BF-A5EO**	65	MALE	Trunk	Alive	T4b	N0	M0	Stage IIC	338
**TCGA-FR-A7UA**	65	FEMALE	Extremities	Alive	T2a	N0	M0	Stage IB	801
**TCGA-FS-A1ZW**	65	MALE	Extremities	Alive	T2b	N1a	M0	Stage IIIB	1505
**TCGA-D9-A149**	65	FEMALE	Trunk	Alive	TX	N1b	M0	Not Available	1513
**TCGA-WE-A8K5**	65	MALE	Extremities	Dead	T2a	N3	M1c	Stage IV	1654
**TCGA-EE-A2GU**	65	FEMALE	Extremities	Alive	T1a	N0	M0	Stage IA	2043
**TCGA-EE-A29T**	51	FEMALE	Trunk	Alive	TX	NX	M0	Not	10523

								Available		
**TCGA-LH-A9QB**	24	FEMALE	Trunk	Alive	Not Available	Not Available	Not Available	Not Available	10860
**TCGA-D3-A8GR**	54	FEMALE	Extremities	Dead	Tis	N0	M0	Stage 0	Not Available
**TCGA-D3-A2J6**	65	MALE	Extremities	Dead	T3b	N0	M0	Stage IIB	Not Available
**TCGA-EB-A85J**	66	FEMALE	Trunk	Alive	T4a	N0	M0	Stage IIB	-6
**TCGA-RP-A690**	66	FEMALE	Not Available	Alive	Not Available	Not Available	Not Available	Not Available	6
**TCGA-EB-A97M**	66	MALE	Trunk	Alive	T4b	N0	M0	Stage IIC	11
**TCGA-FS-A4F8**	52	MALE	Extremities	Dead	T1	N0	M0	Stage I	Not Available
**TCGA-FS-A1Z4**	62	MALE	Extremities	Dead	T1	N0	M0	Stage I	Not Available
**TCGA-EE-A2GO**	66	FEMALE	Extremities	Alive	T3b	N0	M0	Stage II	3101
**TCGA-EE-A2MF**	39	FEMALE	Extremities	Dead	T2	N0	M0	Stage I	Not Available
**TCGA-EE-A20B**	66	FEMALE	Extremities	Alive	T3	N0	M0	Stage II	3335
**TCGA-W3-A828**	66	MALE	Head and Neck	Dead	T3	N0	M0	Stage II	Not Available
**TCGA-EE-A2MC**	73	MALE	Extremities	Dead	T2	N0	M0	Stage I	Not Available
**TCGA-D3-A8GQ**	66	MALE	Trunk	Dead	T3	N0	M0	Stage II	Not Available
**TCGA-EE-A2MM**	63	FEMALE	Trunk	Dead	T2	N0	M0	Stage I	Not Available
**TCGA-EE-A2M7**	66	MALE	Trunk	Dead	T3a	N0	M0	Stage II	Not Available
**TCGA-EE-A2MH**	66	MALE	Trunk	Dead	T4a	N0	M0	Stage III	Not Available
**TCGA-YG-AA3N**	67	MALE	Trunk	Alive	T4b	N0	M0	Stage IIC	201
**TCGA-FS-A4F0**	67	FEMALE	Trunk	Alive	T4a	N0	M0	Stage IIB	2367
**TCGA-EE-A2GN**	67	MALE	Trunk	Dead	T2b	N0	M0	Stage IIA	2767
**TCGA-D9-A4Z5**	68	MALE	Head and Neck	Alive	T4a	N0	M0	Stage IIB	29
**TCGA-DA-A95Y**	68	MALE	Trunk	Dead	T4b	N0	M0	Stage IIC	302
**TCGA-EE-A17X**	54	MALE	Trunk	Dead	T1a	N0	M0	Stage IA	Not Available
**TCGA-GN-A8LN**	68	MALE	Trunk	Alive	T4b	NX	M0	Stage IIC	420
**TCGA-D3-A2JH**	68	MALE	Extremities	Alive	T1b	N0	M0	Stage IB	952
**TCGA-3N-A9WB**	71	MALE	Trunk	Dead	T1a	NX	M0	Stage IA	Not Available
**TCGA-D3-A8GI**	68	MALE	Trunk	Dead	T1a	N0	M0	Stage IA	Not Available

**TCGA-FS-A1ZK**	68	MALE	Head and Neck	Dead	T4	N0	M0	Stage II	Not Available
**TCGA-ER-A19E**	36	FEMALE	Extremities	Dead	T2a	N0	M0	Stage IB	Not Available
**TCGA-GN-A8LL**	68	FEMALE	Extremities	Dead	T4b	NX	M0	Stage IIC	Not Available
**TCGA-EB-A44O**	69	MALE	Trunk	Alive	T4a	N0	M0	Stage IIB	5
**TCGA-DA-A1IB**	69	FEMALE	Extremities	Dead	T2b	N2b	M0	Stage IIIC	73
**TCGA-FR-A3R1**	69	MALE	Extremities	Alive	T4b	N0	M0	Stage IIC	307
**TCGA-D3-A3CC**	69	FEMALE	Other Specify	Alive	T4b	N0	M0	Stage IIC	2168
**TCGA-EE-A17Y**	69	MALE	Trunk	Dead	T3b	N1a	M0	Stage IIIB	Not Available
**TCGA-EB-A82C**	70	FEMALE	Extremities	Alive	T4b	N0	M0	Stage IIC	17
**TCGA-WE-A8JZ**	70	MALE	Extremities	Alive	T4b	N1a	M0	Stage IIIB	731
**TCGA-D9-A6EA**	70	MALE	Trunk	Alive	T4a	N3	M0	Stage IIIC	766
**TCGA-EE-A29Q**	70	FEMALE	Extremities	Dead	T3b	N0	M0	Stage IIB	1136
**TCGA-EE-A2GM**	70	FEMALE	Trunk	Alive	T4b	N0	M0	Stage IIC	2296
**TCGA-FS-A1ZR**	36	MALE	Trunk	Dead	T2	N0	M0	Stage II	Not Available
**TCGA-WE-A8ZM**	70	MALE	Extremities	Alive	TX	N1b	M0	Stage IIIB	2746
**TCGA-EE-A181**	82	FEMALE	Extremities	Dead	T3	N0	M0	Stage II	Not Available
**TCGA-D3-A2JB**	70	FEMALE	Extremities	Dead	Tis	N0	M0	Stage 0	Not Available
**TCGA-GN-A8LK**	70	MALE	Head and Neck	Dead	T1b	NX	Not Available	Stage IB	Not Available
**TCGA-W3-AA1R**	71	MALE	Head and Neck	Dead	T3	N0	M0	Stage II	Not Available
**TCGA-W3-AA1V**	63	MALE	Other Specify	Dead	T3	N0	M0	Stage II	Not Available
**TCGA-FS-A1ZU**	70	FEMALE	Extremities	Dead	T4b	N0	M0	Stage IIC	Not Available
**TCGA-EE-A3JD**	70	MALE	Not Available	Dead	TX	N2b	M0	Stage III	Not Available
**TCGA-EE-A2MQ**	70	FEMALE	Trunk	Dead	T3a	N2a	M0	Stage IIIA	Not Available
**TCGA-EB-A6QY**	71	MALE	Trunk	Alive	T4b	N0	M0	Stage IIC	-5
**TCGA-RP-A694**	71	MALE	Not Available	Alive	TX	NX	M1c	Stage IV	21
**TCGA-EB-A5UL**	71	MALE	Trunk	Alive	TX	N1	M0	Stage III	108
**TCGA-EE-A3AH**	30	MALE	Extremities	Dead	T3b	N0	M0	Stage II	Not Available
**TCGA-GN-A4U9**	71	MALE	Extremities	Dead	T2b	N3	M0	Stage IIIC	384
**TCGA-EE-A2A2**	71	MALE	Trunk	Alive	T4b	N1b	M0	Stage IIIC	743
**TCGA-FS-A1ZY**	71	MALE	Trunk	Dead	T3b	N0	M0	Stage IIB	Not

									Available
**TCGA-EE-A3J5**	71	MALE	Extremities	Dead	T4a	N1	M0	Stage III	Not Available
**TCGA-FS-A1ZH**	71	FEMALE	Extremities	Dead	T3b	N2c	M1c	Stage IV	Not Available
**TCGA-GN-A26D**	72	FEMALE	Extremities	Dead	T4b	N0	Not Available	Stage IIC	1204
**TCGA-ER-A193**	62	MALE	Head and Neck	Dead	T3b	N0	M0	Stage IIB	Not Available
**TCGA-EE-A2MS**	72	MALE	Extremities	Alive	T3a	N0	M0	Stage II	4151
**TCGA-EE-A184**	72	MALE	Extremities	Dead	T2a	N0	M0	Stage IB	Not Available
**TCGA-EE-A3J4**	72	MALE	Extremities	Dead	T3a	N0	M0	Stage II	Not Available
**TCGA-D3-A1Q9**	72	MALE	Extremities	Dead	T4b	N2a	M0	Stage IIIB	Not Available
**TCGA-FS-A1Z3**	72	FEMALE	Not Available	Dead	TX	N0	M1	Stage IV	Not Available
**TCGA-EB-A5SE**	73	MALE	Trunk	Dead	T3b	NX	M0	Stage IIB	0
**TCGA-EB-A42Y**	73	FEMALE	Extremities	Dead	T4b	N0	M0	Stage IIC	6
**TCGA-BF-AAOU**	73	FEMALE	Trunk	Alive	T4b	N0	M0	Stage IIC	13
**TCGA-D3-A1Q3**	64	MALE	Extremities	Dead	T4b	N0	M0	Stage IIC	Not Available
**TCGA-D9-A4Z3**	73	FEMALE	Extremities	Alive	T4b	N1b	M0	Stage IIIC	104
**TCGA-DA-A960**	73	MALE	Trunk	Alive	T3b	N0	M0	Stage IIB	435
**TCGA-D3-A8GM**	73	MALE	Trunk	Dead	T3b	N0	M0	Stage IIB	2897
**TCGA-GF-A769**	39	MALE	Extremities	Dead	T4b	NX	M0	Stage IIC	Not Available
**TCGA-BF-A1PV**	74	FEMALE	Trunk	Alive	T4b	N0	M0	Stage IIC	14
**TCGA-WE-A8K1**	74	MALE	Extremities	Alive	T3b	N3	M0	Stage IIIC	1173
**TCGA-EE-A180**	69	MALE	Trunk	Dead	T4a	N0	M0	Stage III	Not Available
**TCGA-FS-A1ZM**	74	MALE	Extremities	Alive	T2	N2c	M0	Stage III	3080
**TCGA-D9-A6E9**	75	FEMALE	Extremities	Alive	T3a	N1	M0	Stage IIIA	-5
**TCGA-BF-A5EP**	75	FEMALE	Trunk	Alive	T4b	N3	M0	Stage IIIC	11
**TCGA-EE-A3JE**	75	MALE	Trunk	Alive	T3b	N1a	M0	Stage IIIB	1016
**TCGA-YD-A9TA**	75	MALE	Head and Neck	Alive	Not Available	Not Available	Not Available	Not Available	1496
**TCGA-D3-A3CE**	74	FEMALE	Not Available	Dead	T0	N1b	M0	Stage III	Not Available
**TCGA-FS-A4FC**	75	FEMALE	Extremities	Dead	T3a	N0	M0	Stage IIA	1504
**TCGA-D3-A1QB**	75	FEMALE	Not Available	Alive	T0	N2c	M0	Stage III	2512
**TCGA-FS-A1ZJ**	75	FEMALE	Trunk	Dead	T2	N0	M0	Stage I	Not Available

**TCGA-D3-A3MO**	47	MALE	Trunk	Dead	TX	N2c	M0	Stage III	Not Available
**TCGA-D9-A6EG**	56	MALE	Trunk	Dead	T4a	N1	M0	Stage IIIA	Not Available
**TCGA-D3-A2J9**	75	MALE	Trunk;Extremities	Dead	T4b	N3	M0	Stage IIIC	Not Available
**TCGA-D3-A2JG**	30	FEMALE	Extremities	Dead	T3a	N1a	M0	Stage IIIA	Not Available
**TCGA-EB-A6QZ**	76	FEMALE	Trunk	Dead	T3a	N0	M0	Stage IIA	-3
**TCGA-EE-A29A**	68	MALE	Trunk	Dead	T3a	N1a	M0	Stage IIIA	Not Available
**TCGA-EB-A41B**	76	FEMALE	Extremities	Alive	T4b	N0	M0	Stage IIC	3
**TCGA-BF-AAP7**	76	FEMALE	Extremities	Alive	T4b	N0	M0	Stage IIC	15
**TCGA-BF-A5ES**	76	FEMALE	Trunk	Alive	T4b	N0	M0	Stage IIC	19
**TCGA-D3-A3ML**	70	MALE	Head and Neck	Dead	T3a	N2a	M0	Stage IIIA	Not Available
**TCGA-ER-A42H**	76	MALE	Other Specify	Dead	Not Available	Not Available	Not Available	Not Available	Not Available
**TCGA-EB-A3XE**	77	FEMALE	Trunk	Alive	T3a	N0	M0	Stage IIA	0
**TCGA-RP-A693**	77	MALE	Not Available	Alive	TX	NX	M1c	Stage IV	10
**TCGA-D3-A8GP**	77	MALE	Extremities	Alive	T2	N2c	M0	Stage III	4191
**TCGA-ER-A19C**	77	MALE	Trunk	Dead	T2a	NX	M0	Stage I	Not Available
**TCGA-FS-A4F5**	77	FEMALE	Extremities	Dead	T2a	N0	M0	Stage IB	Not Available
**TCGA-EE-A2A0**	77	FEMALE	Extremities	Dead	T3a	N0	M0	Stage IIA	Not Available
**TCGA-ER-A194**	77	MALE	Other Specify	Dead	Not Available	N0	M0	Not Available	Not Available
**TCGA-EB-A5SF**	78	FEMALE	Extremities	Dead	T4b	NX	M0	Stage IIC	0
**TCGA-GF-A6C9**	78	MALE	Not Available	Alive	Not Available	Not Available	Not Available	Stage IIIB	357
**TCGA-EE-A2GR**	78	MALE	Extremities;Extremities	Dead	T4	N0	M0	Stage II	435
**TCGA-FR-A8YC**	78	MALE	Extremities	Dead	T3b	N0	M0	Stage IIB	Not Available
**TCGA-FS-A1ZF**	78	FEMALE	Extremities	Dead	T4b	N0	M0	Stage IIC	Not Available
**TCGA-D3-A2J7**	67	MALE	Trunk	Dead	T3b	N1b	M0	Stage IIIC	Not Available
**TCGA-D3-A2JD**	58	MALE	Extremities	Dead	T4b	N1b	M0	Stage IIIC	Not Available
**TCGA-EE-A29N**	78	MALE	Trunk	Dead	TX	N0	M0	Not Available	Not Available
**TCGA-WE-A8K6**	79	MALE	Trunk	Alive	TX	N1b	M0	Stage IIIB	262

**TCGA-ER-A19A**	79	MALE	Not Available	Alive	TX	N0	M1	Stage IV	2365
**TCGA-ER-A19K**	79	FEMALE	Head and Neck	Dead	T4b	N0	M0	Stage IIC	Not Available
**TCGA-GN-A26C**	77	MALE	Trunk	Dead	T4b	N2b	M0	Stage IIIC	Not Available
**TCGA-D3-A1Q1**	79	FEMALE	Extremities	Dead	T1b	N3	M0	Stage IIIC	Not Available
**TCGA-D9-A1JX**	80	FEMALE	Extremities	Dead	TX	NX	M0	Not Available	195
**TCGA-FS-A4F9**	80	MALE	Trunk	Alive	T4b	N3	M0	Stage IIIC	378
**TCGA-EE-A2GP**	80	MALE	Extremities	Dead	T4b	N1a	M0	Stage IIIB	Not Available
**TCGA-ER-A199**	86	FEMALE	Extremities	Dead	T4b	N3	M0	Stage IIIC	Not Available
**TCGA-EE-A29L**	78	MALE	Extremities	Dead	T4b	N3	M0	Stage IIIC	Not Available
**TCGA-XV-A9W2**	81	MALE	Extremities	Alive	T1	N0	M0	Stage I	12
**TCGA-BF-A3DN**	81	FEMALE	Extremities	Alive	T3b	N3	M0	Stage IIIC	32
**TCGA-D3-A8GC**	48	MALE	Not Available	Dead	TX	N3	M0	Stage IIIC	Not Available
**TCGA-QB-AA9O**	73	MALE	Not Available	Dead	TX	N3	M0	Stage IIIC	Not Available
**TCGA-D3-A2JE**	75	FEMALE	Trunk	Dead	TX	N3	M0	Stage IIIC	Not Available
**TCGA-ER-A19S**	81	FEMALE	Not Available	Alive	Not Available	Not Available	Not Available	Not Available	1133
**TCGA-ER-A19J**	54	MALE	Not Available	Dead	TX	N3	M1	Stage IV	Not Available
**TCGA-DA-A1IC**	81	MALE	Extremities	Dead	T3a	N2c	M0	Stage IIIB	1926
**TCGA-D3-A1Q8**	33	MALE	Not Available	Dead	T0	N3	M1b	Stage IV	Not Available
**TCGA-EE-A20I**	79	MALE	Extremities	Dead	TX	N0	M1c	Stage IV	Not Available
**TCGA-EB-A4P0**	82	MALE	Trunk	Dead	T4b	N0	M0	Stage IIC	-2
**TCGA-3N-A9WC**	82	MALE	Trunk	Alive	T2b	NX	M0	Stage IIA	1856
**TCGA-3N-A9WD**	82	MALE	Other Specify	Dead	T2a	N1a	M0	Stage IIIA	Not Available
**TCGA-FS-A1ZQ**	31	MALE	Extremities	Dead	TX	N0	M0	Not Available	Not Available
**TCGA-ER-A19F**	82	MALE	Extremities	Dead	Not Available	N0	M0	Not Available	Not Available
**TCGA-ER-A195**	46	MALE	Trunk	Dead	TX	N0	M0	Not Available	Not Available
**TCGA-DA-A95V**	83	FEMALE	Head and Neck	Alive	T4b	N0	Not	Stage IIC	1088

							Available		
**TCGA-EE-A2GJ**	83	MALE	Extremities	Alive	T1a	N0	M0	Stage IA	2270
**TCGA-GN-A268**	83	FEMALE	Extremities	Dead	T4a	N0	M0	Stage IIB	Not Available
**TCGA-ER-A19Q**	37	FEMALE	Extremities	Dead	Not Available	N0	M0	Not Available	Not Available
**TCGA-ER-A19P**	47	FEMALE	Extremities	Dead	Not Available	N0	M0	Not Available	Not Available
**TCGA-ER-A197**	83	FEMALE	Other Specify	Dead	T4b	N1a	M0	Stage IIIB	Not Available
**TCGA-BF-A3DL**	84	FEMALE	Extremities	Alive	T3b	N2	M0	Stage IIIB	28
**TCGA-ER-A19H**	40	MALE	Trunk	Dead	Not Available	N0	M0	Not Available	Not Available
**TCGA-EE-A182**	84	FEMALE	Trunk	Dead	T4b	N1b	M0	Stage IIIC	Not Available
**TCGA-WE-A8K4**	85	MALE	Extremities	Alive	T4a	NX	M0	Stage IIB	306
**TCGA-W3-AA1O**	85	MALE	Not Available	Dead	TX	N2	M0	Stage III	Not Available
**TCGA-EE-A29V**	85	MALE	Head and Neck	Dead	T3b	N1b	M0	Stage IIIC	Not Available
**TCGA-EB-A3Y7**	86	FEMALE	Head and Neck	Dead	T3a	N2c	M0	Stage IIIB	0
**TCGA-BF-AAP1**	86	MALE	Extremities	Alive	T4b	N0	M0	Stage IIC	14
**TCGA-DA-A95Z**	87	MALE	Not Available	Alive	TX	N0	M1a	Stage IV	27
**TCGA-EE-A29D**	87	MALE	Head and Neck	Dead	T3b	N1b	M0	Stage IIIC	Not Available
